# Specific targeting of IL-1β activity to CD8^+^ T cells allows for safe use as a vaccine adjuvant

**DOI:** 10.1038/s41541-020-00211-5

**Published:** 2020-07-23

**Authors:** Bram Van Den Eeckhout, Lien Van Hoecke, Elianne Burg, Sandra Van Lint, Frank Peelman, Niko Kley, Gilles Uzé, Xavier Saelens, Jan Tavernier, Sarah Gerlo

**Affiliations:** 1 VIB-UGent Center for Medical Biotechnology, 9052 Ghent, Belgium; 2 Department of Biomolecular Medicine, Ghent University, 9000 Ghent, Belgium; 3 Department of Biomedical Molecular Biology, Ghent University, 9052 Ghent, Belgium; 4 Orionis Biosciences Inc, Waltham, MA 02451 USA; 5 CNRS 5235, University of Montpellier, 34090 Montpellier, France; 6 Department of Biochemistry and Microbiology, Ghent University, 9000 Ghent, Belgium

**Keywords:** Adjuvants, Influenza virus

## Abstract

Annual administration and reformulation of influenza vaccines is required for protection against seasonal infections. However, the induction of strong and long-lasting T cells is critical to reach broad and potentially lifelong antiviral immunity. The NLRP3 inflammasome and its product interleukin-1β (IL-1β) are pivotal mediators of cellular immune responses to influenza, yet, overactivation of these systems leads to side effects, which hamper clinical applications. Here, we present a bypass around these toxicities by targeting the activity of IL-1β to CD8^+^ T cells. Using this approach, we demonstrate safe inclusion of IL-1β as an adjuvant in vaccination strategies, leading to full protection of mice against a high influenza virus challenge dose by raising potent T cell responses. In conclusion, this paper proposes a class of IL-1β-based vaccine adjuvants and also provides further insight in the mechanics of cellular immune responses driven by IL-1β.

## Introduction

Strong and potent T cell responses are essential for protection of the host against both acute and persisting viral infections^1,2^. In the specific case of influenza, higher numbers of pre-existing antiviral CD8^+^ T cells were found to correlate with less severe illness after infection with pandemic H1N1 (pH1N1) virus^3^. Moreover, T cell epitopes of internal viral proteins are strongly conserved across different subtypes of influenza A, which stands in sharp contrast to antibodies that can prevent hemagglutinin-mediated receptor binding or inhibit neuraminidase activity, which typically are highly strain-specific^4–6^. Therefore, induction of robust antiviral T cell responses towards influenza’s “Achilles’ heel” could be the key to a universal influenza vaccine^7^.

Today’s most frequently used vaccine adjuvants are based on aluminum (alum), mostly because they are safe, low in cost and potent in raising neutralizing antibodies. Unfortunately, alum-supplemented vaccines score poorly in inducing antigen-specific CD8^+^ T cell responses^8,9^. In contrast to alum, agonists of pattern-recognition receptors (PRRs), such as monophosphoryl lipid A (MPLA), can potently promote cellular immunity^10^.

One of the PRRs that plays an essential role in connecting innate and adaptive immunity is the NLRP3 inflammasome^11^. NLRP3 assembly can be triggered by a variety of pathogen- and damage-associated molecular patterns, leading to the activation of the cysteine protease caspase-1 and the subsequent cleavage of immature pro-interleukin-1β (IL-1β) to active IL-1β, which can ultimately be released by different innate immune cells^12^. Ever since the late 1970s, IL-1β is known for its T cell stimulatory properties^13–15^, but only recently it was demonstrated that direct action of IL-1β on CD8^+^ and CD4^+^ T cells promotes their activation, proliferation, and memory development^16–20^. During influenza A virus infection, activation of the NLRP3 inflammasome followed by IL-1β release was found to be critical for the formation of an antiviral CD8^+^ T cell response^21–23^. Moreover, mucosal vaccination of mice with IL-1β as adjuvant protected against infections with homo- and heterologous influenza strains by development of effective CD4^+^ and CD8^+^ tissue-resident memory T cells (T_RM_)^24^.

Although these findings indicate that inflammasome activation is essential for development of effective immunity against influenza virus infection, excessive inflammasome activation and local or systemic IL-1β administration elicit undesired side effects, including fever, neutrophilia and the release of acute phase proteins^25–27^. These cytokine-associated toxicities result from the ubiquitous expression of the IL-1 receptor (IL-1R) complex in the host and have hampered the clinical development of IL-1β as an adjuvant^28^.

To safely exploit the clinical potential of toxic cytokines, we recently developed the AcTakine (Activity-on-Target cytokine) concept^29^. Here, a cytokine mutant with a strongly reduced biological activity is genetically coupled to a single-domain antibody (sdAb), which binds to a cell type-specific surface molecule. This allows the cytokine to remain inactive en route through the body to only reveal its full agonistic activity upon target cell binding. Earlier, we demonstrated that AcTakines based on type I interferon (IFN), targeted to type I cross-presenting dendritic cells (DCs)^30^ or tumor cells^31^, induce tumor eradication in mice without systemic side effects. More recently, we showed that treatment with a tumor necrosis factor (TNF)-based AcTakine targeted to CD13 allows for destruction of large established tumors without detectable toxicity in vivo^32^.

In this study, we developed an AcTakine that targets an IL-1β mutant to CD8^+^ T cells for use as a vaccine adjuvant (from now on referred to as CD8α AcTaleukin-1/ALN-1). In vivo, CD8α ALN-1 potently promotes the CD8^+^ T cell response to antigen, with a significantly reduced toxicity profile compared with wild-type (WT) IL-1β. Inclusion of CD8α ALN-1 in a prophylactic H3N2-based influenza vaccine fully protects mice against challenge infection with a high dose of a heterosubtypic pH1N1 influenza A virus, which correlated with the induction of strong, functional and long-lasting antiviral T cell responses. Moreover, this work provides further insights in cellular immune responses driven by vaccination with IL-1β.

## Results

### Q148G is an IL-1β mutant with strongly reduced biological activity that can be completely reactivated upon targeting using a CD8α sdAb

**Fig. 1:**
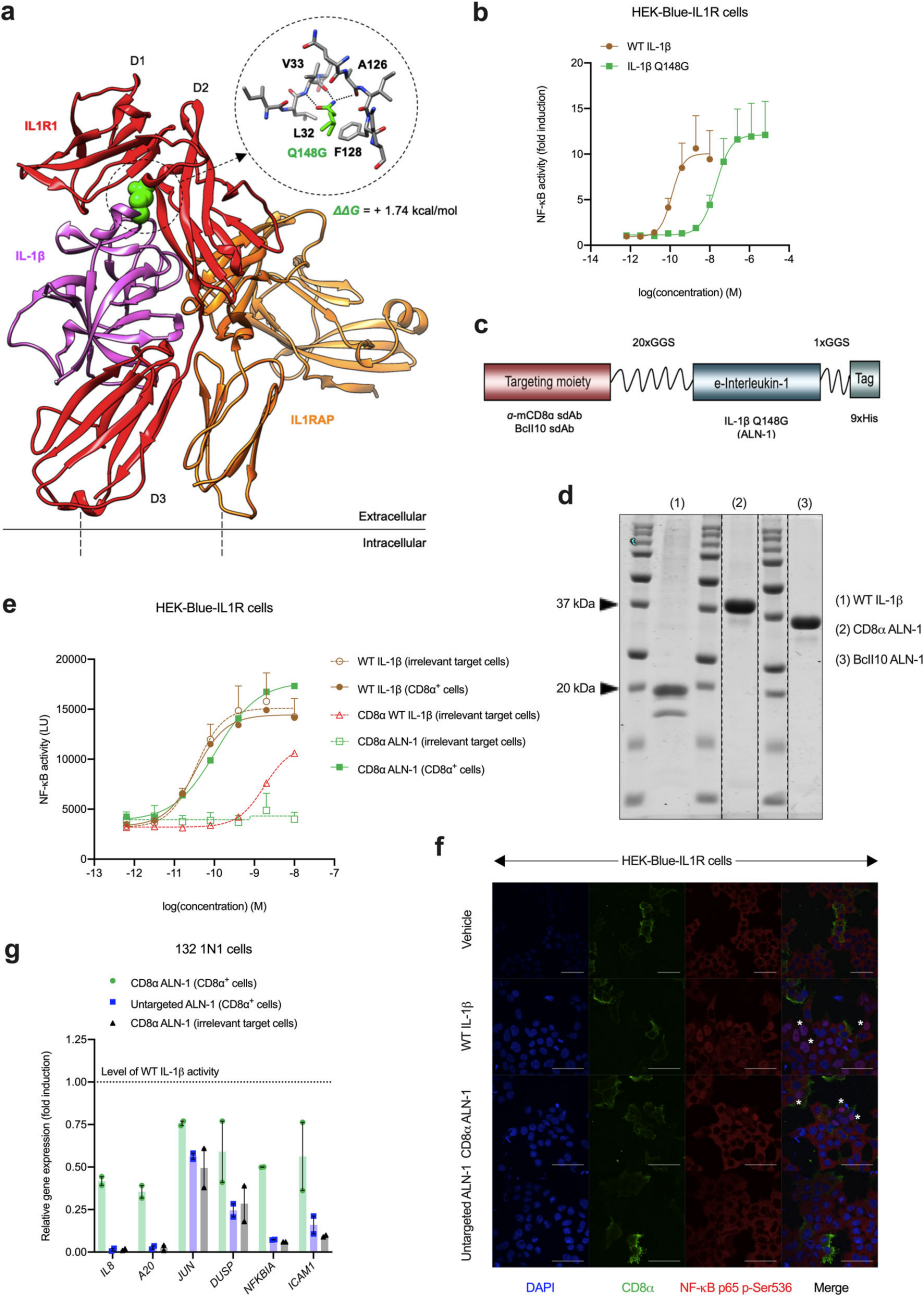
Q148G is an IL-1β mutant with strongly reduced biological activity that can be completely reactivated upon targeting using a CD8α sdAb. **a** Model of IL-1R1 (red, domains D1–D3) and IL-1RAP (orange) binding to IL-1β (purple), showing a detail of the Q148G mutation. **b** NF-κB-driven luciferase reporter gene expression in HEK-Blue-IL1R cells. NF-κB activity is normalized to background and expressed as fold induction. Data points represent the mean of four independent experiments ± s.e.m. **c** CD8α ALN-1 design: IL-1β Q148G is coupled to an anti-CD8α sdAb by a 20xGGS linker. The fusion to the BcII10 sdAb is used as an untargeted control. **d** Representative SDS-PAGE protein gel demonstrating purity after recombinant production in HEK-293-F cells. Dotted lines indicate nonadjacent lanes from the original gel. **e** NF-κB-driven luciferase reporter gene expression in HEK-Blue-IL1R cells, transiently transfected with CD8α or irrelevant target DNA. NF-κB activity is expressed as absolute luminescence units (LU). Data points represent the mean of at least five independent experiments ± s.e.m. **f** Confocal microscopy showing nuclear translocation of NF-κB p65 in HEK-Blue-IL1R cells (1:1 mix of cells transfected with CD8α or irrelevant target DNA). Stimulation with vehicle, WT IL-1β, CD8α ALN-1, or untargeted ALN-1 (0.5 nM). The scale bar (white) indicates 50 μm. Asterisk (*) indicates cells of particular interest. **g** RT-qPCR analysis showing expression of NF-κB-, p38 MAPK-, and AP-1-driven target genes in human 132 1N1 astrocytes, transfected with CD8α or irrelevant target DNA. Stimulation with WT IL-1β, CD8α ALN-1, or untargeted ALN-1 (0.5 nM). Data are normalized to gene expression upon WT IL-1β stimulation. Bars represent mean ± s.e.m. of two independent experiments. See also Supplementary Fig. 1.

Based on a model of IL-1β bound to its receptors (Fig. 1a), we generated several human IL-1β mutants, predicted to have reduced biological activity. Of these, the IL-1β mutant Q148G (Fig. 1a: detail) was selected for further study. Q148 is an important residue for IL-1R1 binding, located in one of two known cytokine-receptor contact areas. Within this interface, 118 Å^2^ of the amino acid’s accessible surface becomes buried while it makes multiple connections with the IL-1R1: two direct contacts with F128 and L32 and three hydrogen bonds with the backbone amides of V33 and A126 of the receptor subunit (Fig. 1a: detail)^33^. Mutating the glutamine at position 148 to glycine destabilizes the interaction with 1.74 kcal/mol, as predicted by Fold-X. This leads to an ~168-fold reduction in activity compared with WT IL-1β (Fig. 1b), as measured by NF-κB-driven expression of a luciferase reporter in HEK-293 cells stably transfected with the mouse IL-1R complex (HEK-Blue-IL1R).

To locally reactivate IL-1β Q148G by targeting to cell type-specific surface molecules, we genetically fused the mutant cytokine to a sdAb that is specific for mouse CD8α^34^, thereby creating the CD8α ALN-1 (Fig. 1c). This sdAb was found not to impede the activation of CD8^+^ T cells during antigen presentation in vitro (Supplementary Fig. 1a). This is important, as different anti-CD8 antibodies with the potential to interfere with CTL activation have been described before^35^. As a negative control for targeting, we made a genetic fusion with a BcII10 sdAb, which is directed against bacterial β-lactamase^36^. These fusion proteins were produced in mammalian HEK-293-F cells and subsequently purified using the built-in 9xHis-tag (Fig. 1d and Supplementary Fig. 1b). We measured an ~60-fold reduction in the biological activity of WT IL-1β after N-terminal coupling to the CD8α sdAb (Fig. 1e), meaning that the activity of CD8α ALN-1 is ~9000-fold lower than that of WT. Next, we evaluated CD8α ALN-1’s reactivation-by-targeting on cells transiently transfected with CD8α. We found that the agonistic activity of CD8α ALN-1 could be fully restored up to WT level on CD8α^+^ cells, while CD8α ALN-1 remained completely inactive on cells lacking the sdAb target. This was demonstrated by measuring NF-κB activation via reporter gene (Fig. 1e) and confocal microscopy (Fig. 1f) experiments in HEK-Blue-IL1R cells. Nuclear translocation of the NF-κB p65 subunit upon CD8α ALN-1 treatment occurred only in CD8α^+^ cells, whereas WT IL-1β induced NF-κB activation independently of CD8α expression and untargeted ALN-1 did not show any activity. In addition, we evaluated IL-1R signaling further downstream by measuring the expression of selected NF-κB, p38 MAPK, and AP-1 target genes via RT-qPCR analysis in human 132 1N1 astrocytes, which endogenously express the IL-1R complex and were transiently transfected with CD8α or an irrelevant target protein. We found that the induction of most genes (*IL8*, *A20*, *NFKBIA*, and *ICAM1*) was only restored upon targeting of CD8α ALN-1 to CD8α^+^ cells (Fig. 1g and Supplementary Fig. 1c). The expression of *JUN* and *DUSP* was restored as well, but a higher background activity of the untargeted ALN-1 was apparent for these transcripts. Importantly, we found that the biological activity of CD8α ALN-1 upon targeting is dependent on the level of target antigen expression (Supplementary Fig. 1d, e).

Altogether, these findings illustrate that Q148G, an IL-1β mutant with strongly reduced biological activity, can regain WT activity upon targeting with a CD8α-specific sdAb.

### CD8α ALN-1 promotes antigen-dependent activation and proliferation of CD8^+^ T cells in vitro

**Fig. 2:**
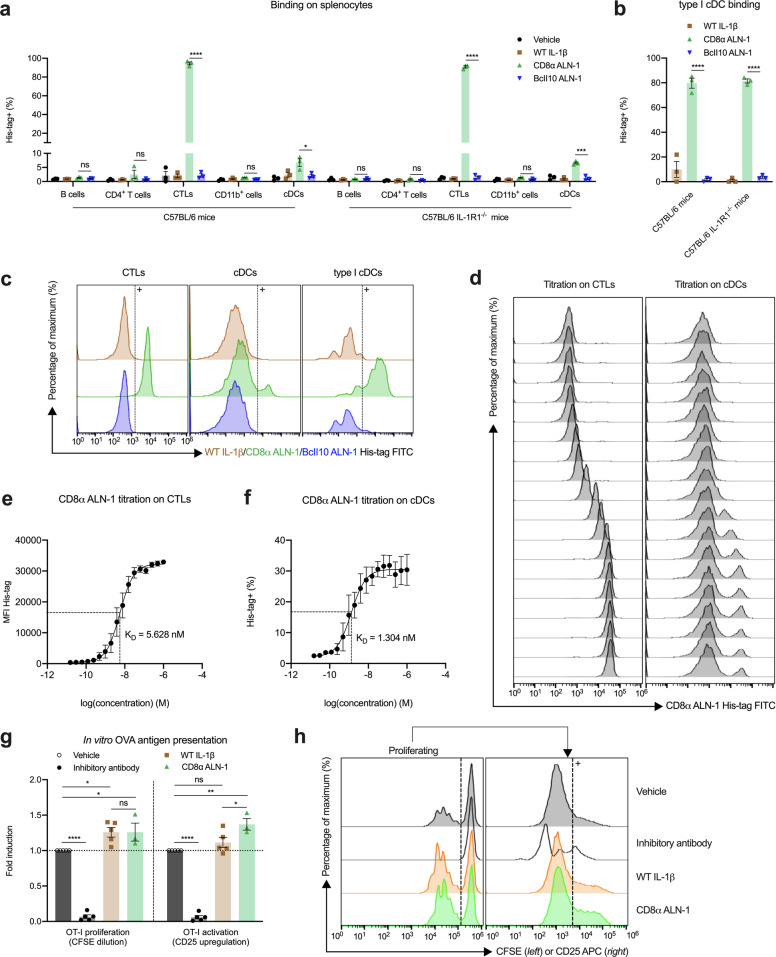
CD8α ALN-1 promotes antigen-dependent activation and proliferation of CD8^+^ T cells in vitro. **a** Flow cytometry analysis of in vitro binding within different immune cell populations of C57BL/6 (IL-1R1^−/−^) mouse spleens. **b** Flow cytometry analysis of in vitro binding within the XCR1^+^ cDC population of C57BL/6 (IL-1R1^−/−^) mouse spleens. Binding of vehicle, WT IL-1β, CD8α ALN-1, and untargeted BcII10 ALN-1 at 1 nM in **a** or 10 nM in **b**. Bars represent the mean ± s.e.m. of three independent experiments. **c** Representative flow cytometry histograms demonstrating binding within the CTL, cDC, and type I cDC subset. **d** Representative flow cytometry histograms of CD8α ALN-1 titration on CTLs and cDCs. Concentration range shown in **e** and **f**. Quantification of CD8α ALN-1 binding affinity on CTLs (**e**) and cDCs (**f**). Data points represent the mean ± s.e.m. of three independent experiments. **g** Flow cytometry analysis of OT-I proliferation (left) and activation (right) in in vitro OT-I cocultures. Stimulation with vehicle, inhibitory antibody, WT IL-1β, or CD8α ALN-1 (1 nM). Bars represent the mean ± s.e.m. of at least three independent experiments. Fold induction was calculated as CFSE dilution or CD25 upregulation under treatment conditions relative to vehicle. **h** Representative flow cytometry histograms of OT-I proliferation (left) and CD25 upregulation in the divided OT-I subset (right). ****p < 0.0001; ***p < 0.001; **p < 0.01; *p < 0.05; ns, *p* ≥ 0.05 by unpaired Student’s *t* test (two-tailed) in **a** and **b** or by one-way ANOVA with Tukey’s multiple comparisons test in **g**. See also Supplementary Figs. 2 and 3.

We further evaluated the specificity and affinity of CD8α ALN-1 for CD8^+^ cells in vitro using flow cytometry (gating strategies in Supplementary Fig. 2a–d). CD8α ALN-1 binds two different cellular subsets of murine splenocytes: CD4^−^ T cells, corresponding to cytotoxic T lymphocytes (CTLs), and conventional DCs (cDCs) (Fig. 2a, c). Furthermore, the cDCs bound by CD8α ALN-1 expressed XCR1, identifying them as type I cDCs, which are known to be CD8α^+^ in mice^37^ (Fig. 2b, c). We did not observe binding of CD8α ALN-1 to any other immune cell type tested (Fig. 2a), including NK cells (Supplementary Fig. 2e). No binding could be detected for WT IL-1β and untargeted BcII10 ALN-1 (Fig. 2a–c and Supplementary Fig. 2e). The importance of this sdAb for specific cell targeting is confirmed by the observation that CD8α ALN-1 binding remained intact on IL-1R1^−/−^ splenocytes (Fig. 2a, b). Titration of the CD8α sdAb on the CTL and cDC subsets showed that this targeting moiety binds with nanomolar affinity (*K*_D_ of 5.6 nM for CTL binding and 1.3 nM for cDC binding) (Fig. 2d–f).

Due to the cross-reactivity of human IL-1β in mouse^38^, we could use the murine OT-I coculture system to address the potential adjuvant capacity of CD8α ALN-1 in vitro. We found that, despite high background proliferation of antigen-exposed OT-I cells, CD8α ALN-1 (like WT IL-1β) further promoted SIINFEKL peptide-dependent proliferation of OT-I cells (Fig. 2g–h, left; gating strategy in Supplementary Fig. 3a). This effect completely depended on presentation of antigen by bone marrow-derived DCs (BM-DCs) to OT-I cells (Supplementary Fig. 3b). Similar results were obtained using IL-1R1^−/−^ BM-DCs in the cocultures, suggesting that CD8α ALN-1 acts directly on the antigen-specific CTLs (Supplementary Fig. 3c). Moreover, treatment with CD8α ALN-1 led to an enhanced upregulation of CD25 (IL-2Rα) in the dividing OT-I cell subset (Fig. 2g, h, right) and augmented release of the effector cytokines IFN-γ and TNF, indicative for enhanced CTL activation (Supplementary Fig. 3d)^39^.

In conclusion, we demonstrated that CD8α ALN-1 can efficiently deliver IL-1β activity to CD8^+^ T cells, leading to a moderately enhanced antigen-specific T cell response in vitro.

### CD8α ALN-1 promotes CD8^+^ T cell proliferation and effector functions in response to antigen in vivo

**Fig. 3:**
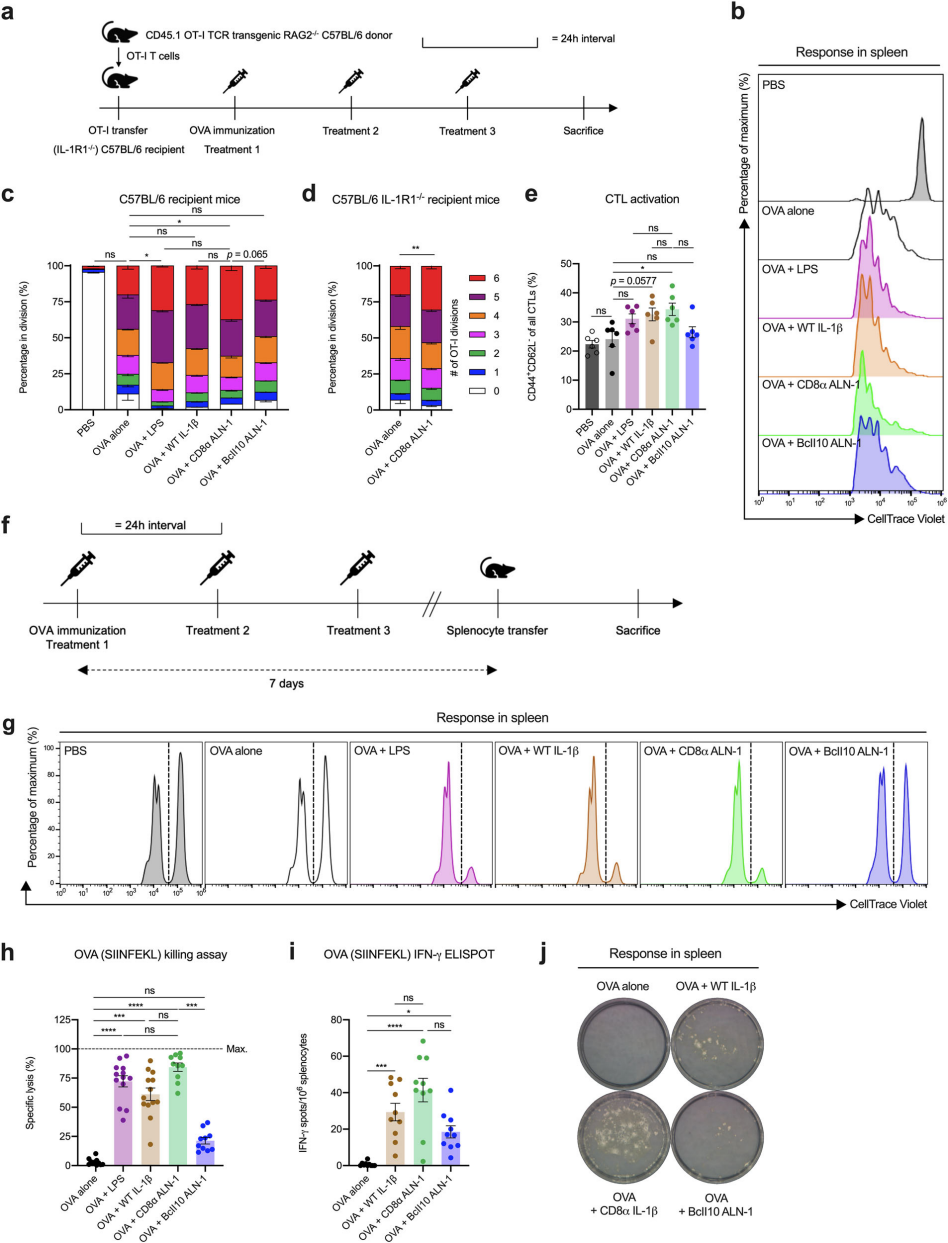
CD8α ALN-1 promotes CD8^+^ T cell proliferation and effector functions in response to antigen in vivo. **a** OT-I cells were transferred i.v. in C57BL/6 (IL-1R1^−/−^) mice and OVA (100 μg) was delivered i.p. 1 day later or not (PBS). OVA-immunized mice received i.p. treatments with PBS, LPS (25 μg), WT IL-1β (5 μg), CD8α ALN-1, or untargeted BcII10 ALN-1 (10 μg) every 24 h for 3 days. Flow cytometry was performed 1 day after the last treatment. **b** Representative flow cytometry histograms showing OT-I proliferation in C57BL/6 recipient mice. Stacked histograms summarizing OT-I proliferation in C57BL/6 (**c**) or C57BL/6 IL-1R1^−/−^ (**d**) recipient mice. Individual stacks represent the mean percentages of proliferating OT-I cells in certain stages of cell division ± s.e.m. Shown is a pool of two independent experiments with *n* = 6 (**c**) or 10 (**d**) mice/group combined. **e** CD44/CD62L expression on CD8^+^ T cells (spleen) from C57BL/6 recipient mice. Bars represent the mean ± s.e.m. of a pool of two independent experiments with *n* = 6 mice/group combined. **f** Mice were treated as described in **a**. One week after OVA delivery, a mixed splenocyte pool (CTV^high^ labeled/SIINFEKL-loaded and CTV^low^ labeled/nonloaded) was i.v. transferred. Flow cytometry was performed 1 day later. Representative flow cytometry histograms illustrating SIINFEKL-directed killing in C57BL/6 recipient mice (**g**) and quantification (**h**). Bars represent the mean ± s.e.m. of a pool of two independent experiments with *n* = 13 (OVA alone, OVA + LPS and OVA + WT IL-1β) or 10 (OVA + CD8α ALN-1 and OVA + BcII10 ALN-1) mice/group combined. **i** CTL responses against SIINFEKL were measured in spleens by IFN-γ ELISPOT 1 week after OVA immunization. Bars represent the mean ± s.e.m. of a pool of two independent experiments with *n* = 10 mice/group combined. **j** Representative ELISPOT pictures, depicting numbers of spots after different treatments. ****p < 0.0001; ***p < 0.001; **p < 0.01; *p < 0.05; ns, *p* ≥ 0.05 by Kruskal–Wallis test with Dunn’s multiple comparisons test in **c**, **h**, and **i** or by unpaired Mann–Whitney *U* test (two-tailed) in **d** or by one-way ANOVA with Tukey’s multiple comparisons test in **e**. See also Supplementary Figs. 4 and 5.

To investigate whether CD8α ALN-1 displays cellular adjuvant activity in vivo, as was earlier reported for WT IL-1β^16^, we first performed OT-I adoptive transfer experiments (Fig. 3a; gating strategy in Supplementary Fig. 4a). In this model, intraperitoneal (i.p.) immunization of mice with OVA alone already resulted in the proliferation of OT-I cells compared with mice treated without antigen (PBS) (Fig. 3b, c). Coadministration of OVA and LPS (used as a positive control adjuvant) further increased OT-I division. Characteristic for this effect is the significant increase in the fraction of OT-I cells in the latest stage of cell proliferation (i.e., the sixth CellTrace Violet (CTV) dilution peak) compared with OVA immunization alone. Similar to the effect of LPS, CD8α ALN-1 significantly enhanced OVA-induced OT-I proliferation. No significant effect of untargeted BcII10 ALN-1 was observed when compared with OVA immunization alone. When using C57BL/6 IL-1R1^−/−^ recipient mice, the observed CD8α ALN-1 effect on proliferation remained intact (Fig. 3d), suggesting that CD8α ALN-1 acts directly on the OT-I cells. We found that treatment with OVA and CD8α ALN-1 increased the fraction of OT-I cells within the total CD8^+^ T cell population and the absolute numbers of OT-I cells both in lymphoid (lymph nodes (LNs) and spleen) and peripheral (liver and lungs) organs compared with delivery of OVA alone (Supplementary Fig. 4b–e). Moreover, CTL activation was enhanced in the spleens of mice treated with OVA and CD8α ALN-1 compared with mice immunized with OVA alone, as indicated by the simultaneous upregulation of CD44 and downregulation of CD62L (Fig. 3e; representative dot plots in Supplementary Fig. 4f)^40^.

We next explored whether CD8α ALN-1 could also boost endogenous OVA-specific CD8^+^ T cell activity using an in vivo killing assay (Fig. 3f; gating strategy and splenocyte labeling in Supplementary Fig. 5a, b). No antigen-specific target cell killing was observed in mice immunized with OVA alone, while cytolytic activity was strongly promoted upon coadministration of LPS, WT IL-1β, and CD8α ALN-1 (Fig. 3g, h). In this assay, CD8α ALN-1 was found to be equally efficacious as high-dose LPS and WT IL-1β. Conversely, treatment with OVA and untargeted BcII10 ALN-1 had no significant effect on target cell killing compared with OVA immunization alone.

CD8^+^ T cells isolated from mice that were treated with OVA and WT IL-1β or CD8α ALN-1 released more IFN-γ upon ex vivo restimulation with SIINFEKL, as measured by enzyme-linked immunospot (ELISPOT) (Fig. 3i, j). In agreement with the results from the in vivo killing experiment, the effect of CD8α ALN-1 was comparable to that of WT IL-1β. Some residual activity of the combination of OVA with untargeted BcII10 ALN-1 was apparent in the ELISPOT.

These data demonstrate that CD8α ALN-1 stimulates antigen-specific CTL responses in vivo.

### Systemic treatment of mice with CD8α ALN-1 shows an improved safety profile compared with WT IL-1β

**Fig. 4:**
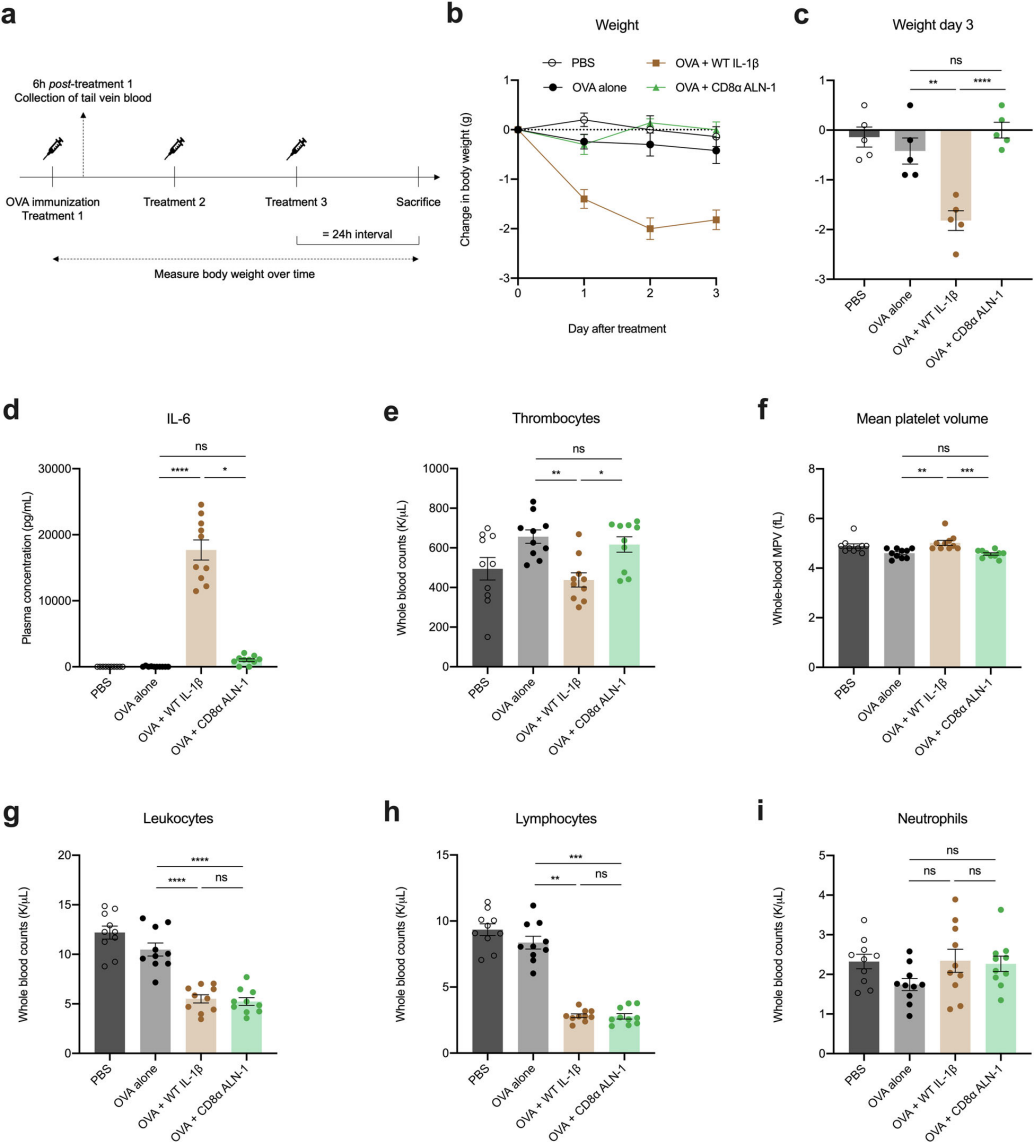
Systemic treatment of mice with CD8α ALN-1 shows an improved safety profile compared with WT IL-1β. **a** C57BL/6 mice received one i.p. OVA (100 μg) administration together with i.p. treatments with WT IL-1β (5 μg) or CD8α ALN-1 (10 μg) every 24 h for 3 days. Controls include mice treated with PBS or OVA alone. Tail vein blood was sampled 6 h after the first treatment and body weight was measured over time. Change in body weight over time (**b**) or after 3 days of treatment (**c**). Data points (**b**) and bars (**c**) represent the mean ± s.e.m. of a representative of two independent experiments with *n* = 5 mice/group. Hematological analysis of fresh EDTA-treated blood (**e**–**i**) or plasma derived from this blood (**d**), showing systemic IL-6 levels (**d**), platelet counts (**e**) and mean platelet volumes (**f**), total white blood cell counts (**g**), lymphocyte counts (**h**), and neutrophil counts (**i**). Bars represent the mean ± s.e.m. of a pool of two independent experiments with *n* = 10 mice/group combined. ****p < 0.0001; ***p < 0.001; **p < 0.01 *p < 0.05; ns, *p* ≥ 0.05 by one-way ANOVA with Tukey’s multiple comparisons test (**c**, **g**, **i**) or by Kruskal–Wallis test with Dunn’s mult**i**ple comparisons test (**d**–**f**, **h**). See also Supplementary Fig. 6.

Because WT IL-1β is well known for its capacity to induce systemic inflammation, we addressed the safety of the immunization strategy described above (Fig. 4a). As a readout of morbidity, we measured mouse body weight over time and evaluated the systemic release of IL-6 in blood, sampled 6 h after the first treatment. In these samples, we also looked for abnormalities in different hematological parameters. We found that repeated delivery of WT IL-1β leads to detrimental side effects, such as severe body weight loss (Fig. 4b, c), systemic release of IL-6 (Fig. 4d) and platelet destruction (Fig. 4e, f), whereas treatment with a corresponding dose of CD8α ALN-1 had no significant effects on these parameters. Intriguingly, both WT IL-1β and CD8α ALN-1 elicit leukopenia (Fig. 4g), which we found is primarily due to a reduction in circulating lymphocytes (Fig. 4h) as the level of neutrophils remains unaltered (Fig. 4i).

We looked further into this leukopenia and observed that, based on Hemavet 950FS measurements, this decrease in circulating lymphocytes is apparent 6 h after treatment and is thereafter further maintained when CD8α ALN-1 is repeatedly administered (Supplementary Fig. 6a). Using flow cytometry, we further investigated which lymphocyte subpopulations were exactly affected (see Supplementary Fig. 6b for the gating strategy) and observed drops in absolute counts for all evaluated cell types (Supplementary Fig. 6c).

Together, these data show that overall well-being of mice is improved when IL-1β activity is restricted to CD8^+^ T cells. CD8α ALN-1 administration does, however, impact lymphocyte homeostasis or redistribution.

### An influenza vaccine adjuvanted with CD8α ALN-1 protects mice against viral infection

**Fig. 5:**
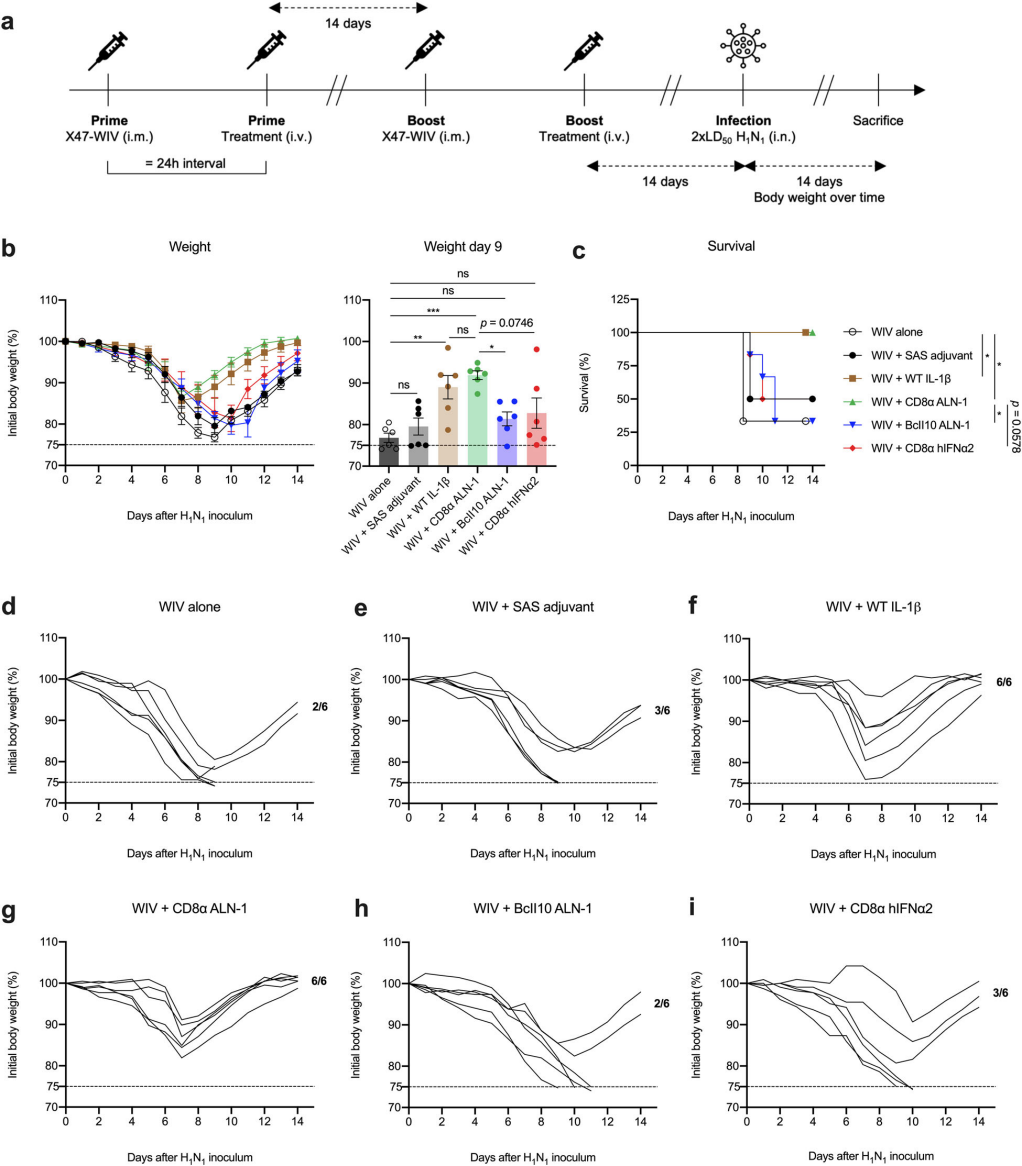
An influenza vaccine adjuvanted with CD8α ALN-1 protects mice against viral infection. **a** BALB/c mice were vaccinated i.m. with X47-WIV (15 μg), either alone or combined with SAS adjuvant (15 μg i.m. together with WIV), WT IL-1β (5 μg), CD8α ALN-1, untargeted BcII10 ALN-1, or CD8α hIFNα2 (10 μg i.v. 24 h post WIV). An identical boost treatment was delivered 2 weeks later. A heterosubtypic pH1N1 virus that shares strongly conserved T cell epitopes with X47-WIV was used to infect the mice 2 weeks later (i.n. 2 × LD_50_). **b** Change in body weight over time (left) or after nine days of infection (right) of mice challenged i.n. with a high inoculum (2 × LD_50_) of pH1N1 influenza virus. Data points (left) or bars (right) represent the mean ± s.e.m. of a representative of two independent experiments with *n* = 6 mice/group. **c** Kaplan–Meier curve representing survival of mice during pH1N1 influenza virus infection. Data points represent the mean survival (%) ± s.e.m. of a representative of two independent experiments with *n* = 6 mice/group. ***p < 0.001; **p < 0.01; *p < 0.05; ns, *p* ≥ 0.5 by one-way ANOVA with Tukey’s multiple comparisons test in **b** or by log-rank testing in **c**. Change in body weight (%) over time of virus-infected mice vaccinated with WIV alone (**d**) or combined with either SAS (**e**), WT IL-1β (**f**), CD8α ALN-1 (**g**), untargeted BcII10 ALN-1 (**h**), and CD8α hIFNα2 (**i**).

Adjuvants promoting T cell responses against conserved influenza epitopes could be critical in the search for a universal influenza vaccine^1,4,41^. Therefore, we evaluated the efficacy of CD8α ALN-1 as an adjuvant in a prime-boost vaccination strategy, using whole-inactivated X47 (H3N2) virus (WIV) as source of viral antigen, to protect against challenge infection with a heterosubtypic H1N1 2009 pandemic (pH1N1) influenza virus, a mouse-adapted derivative from a clinical isolate responsible for the 2009 flu pandemic (Fig. 5a). Protection in this model is expected to depend predominantly on T cell responses as mainly T cell epitopes are conserved between heterosubtypic X47 and pH1N1^42^. We found that all mice vaccinated with CD8α ALN-1 survived the challenge infection with pH1N1 (Fig. 5b–i). Treatment with WIV and CD8α ALN-1 was as efficacious as treatment with WIV and WT IL-1β. Four out of six mice immunized with WIV alone succumbed to the infection. This suggests that CD8α ALN-1 treatment induces potent cross-protective antiviral immunity. Interestingly, vaccination with CD8α ALN-1 also outperforms the commercial Sigma Adjuvant System (SAS), an oil-in-water emulsion containing MPLA, for which no significant difference with WIV alone could be observed. Moreover, no protection compared with the WIV only vaccine was observed when using untargeted BcII10 ALN-1 or a CD8α sdAb coupled to human IFNα2. As human IFNα2 is inactive in mice and has a molecular weight comparable to WT IL-1β, this molecule is used as control for possible effects via the CD8α sdAb.

Altogether, these data show that CD8α ALN-1 can be used as an adjuvant to protect against influenza A virus infection by prime-boost vaccination.

### The protective antiviral effect of CD8α ALN-1 correlates with the induction of strong and long-lasting influenza-specific T cell responses in lung and lymphoid tissues

Influenza virus nucleoprotein (NP) is a conserved internal antigen, known for its ability to mount strong and long-lasting T cell responses^43^. Functionally, NP is an RNA-binding protein that encapsulates the viral genome and is required for RNA transcription, replication, and viral genome packaging^44^.

**Fig. 6:**
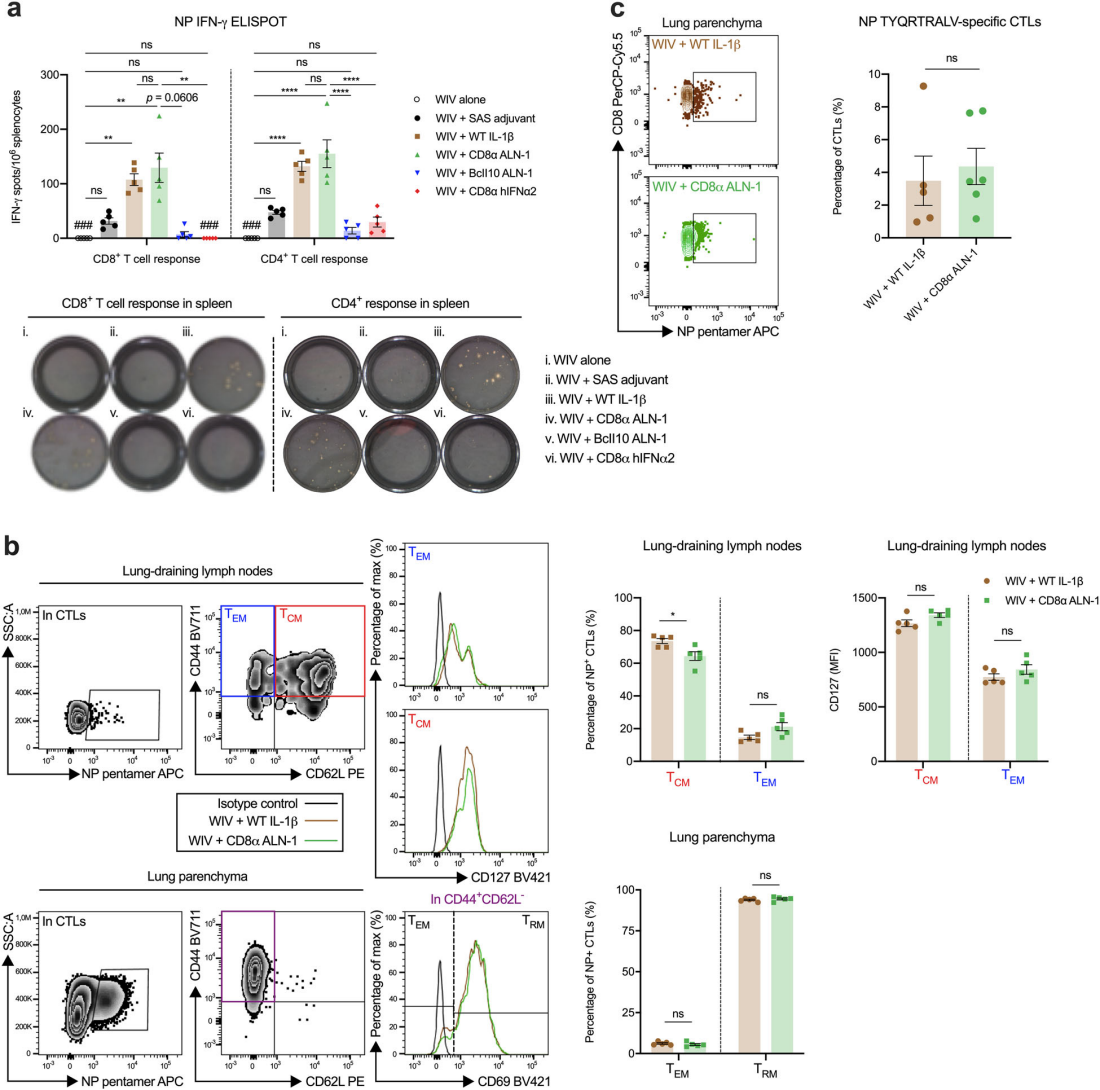
The protective antiviral effect of CD8α ALN-1 correlates with the induction of strong and long-lasting influenza-specific T cell responses in lung and lymphoid tissues. **a** Mice were vaccinated as described in Fig. 5a. Two weeks post boost, NP-specific CD8^+^ and CD4^+^ T cell responses in spleens of vaccinated mice were measured by IFN-γ ELISPOT. Representative ELISPOT pictures are shown, depicting numbers of spots after different vaccinations. Bars represent the mean ± s.e.m. of a representative of two independent experiments with *n* = 5 mice/group. Undetectable responses are indicated by triple hash symbol (###). **b** Detection and phenotyping of NP-specific CD8^+^ T cells in the lung-draining LNs and lung parenchyma of mice vaccinated with WIV and WT IL-1β or CD8α ALN-1, 7 days post pH1N1 influenza A virus infection. Bars represent the mean ± s.e.m. of a representative of two independent experiments with *n* = 5 mice/group. **c** Flow cytometric detection of NP-specific CD8^+^ T cells in the lungs of surviving mice that were vaccinated with WIV and WT IL-1β or CD8α ALN-1, 50 days post pH1N1 influenza A virus infection. Representative dot plots showing the fraction of NP-pentamer^+^ cells in the CTL population. Bars represent the mean ± s.e.m. of one experiment with *n* = 5 (WT IL-1β) or 6 (CD8α ALN-1) mice/group. ****p < 0.0001; **p < 0.01; *p < 0.05; ns, *p* ≥ 0.05 by Kruskal–Wallis test with Dunn’s multiple comparisons test in (**a**, CD8 ELISPOT) or by one-way ANOVA with Tukey’s multiple comparisons test (**a**, CD4 ELISPOT) or by unpaired Student’s *t* test (two-tailed) in **b** and **c**. See also Supplementary Fig. 7.

We sampled mice on different time points before, during and after pH1N1 virus encounter to evaluate whether vaccination with WIV combined with CD8α ALN-1 induces NP-specific T cell responses. Two weeks after the boost, we observed a significantly stronger increase in the number of splenic NP-specific IFN-γ producing CD8^+^ and CD4^+^ T cells in mice that had been vaccinated with WIV and CD8α ALN-1 compared with mice immunized with WIV alone (Fig. 6a). CD8α ALN-1 proved to be as efficacious as WT IL-1β to induce these responses. The combinations of WIV with either SAS, untargeted BcII10 ALN-1 or CD8α hIFNα2 had no significant effect, which is consistent with their inability to enhance survival.

We further characterized the NP-specific CD8^+^ T cells, induced upon vaccination with both WT IL-1β and CD8α ALN-1, in lung-draining mediastinal LNs and lung parenchyma of mice, 1 week post influenza A virus challenge using flow cytometry (Fig. 6b; gating strategy in Supplementary Fig. 7a, b)^45^. In the draining LNs, the bulk of these NP-specific CD8^+^ T cells showed a CD62L^+^CD44^+^ expression pattern, representing a central memory T cell (T_CM_) pool. A much smaller fraction consisted of CD62L^−^CD44^+^ cells, which identify as effector memory T cells (T_EM_). Consistent with this profile, higher expression of the prosurvival marker CD127 (IL-7Rα) was found in the T_CM_ subset compared with the T_EM_ cells. In the functional lung parenchyma, most of the retrieved NP-specific CD8^+^ T cells were positive for the residency marker CD69, which is indicative for a T_RM_ phenotype. A smaller proportion of antiviral CTLs in the lung were CD69^−^ T_EM_ cells^45,46^. Moreover, we found that NP-specific CD8^+^ T cells were still present in the lungs of mice vaccinated with WT IL-1β and CD8α ALN-1 by day 50 after pH1N1 infection (Fig. 6c; gating strategy in Supplementary Fig. 7c).

### The transcriptional landscape of CD8^+^ T cells isolated from vaccinated mice during influenza virus infection supports the cellular adjuvant effect of CD8α ALN-1

Thus far, our data clearly demonstrate that vaccination of mice with WIV and CD8α ALN-1 raises potent and long-lasting CD8^+^ and CD4^+^ T cell responses, directed against the viral NP antigen. In order to better understand how selective IL-1β activity on CD8^+^ T cells establishes these effects, we performed RNA sequencing and looked into transcriptomic changes in CTLs. For this, we sorted CD8^+^ T cells from the lung parenchyma and lung-draining mediastinal LNs of mice vaccinated with either WIV alone or combined with WT IL-1β or CD8α ALN-1, 1 week after viral challenge (see Supplementary Fig. 8a, b for the gating strategy).

**Fig. 7:**
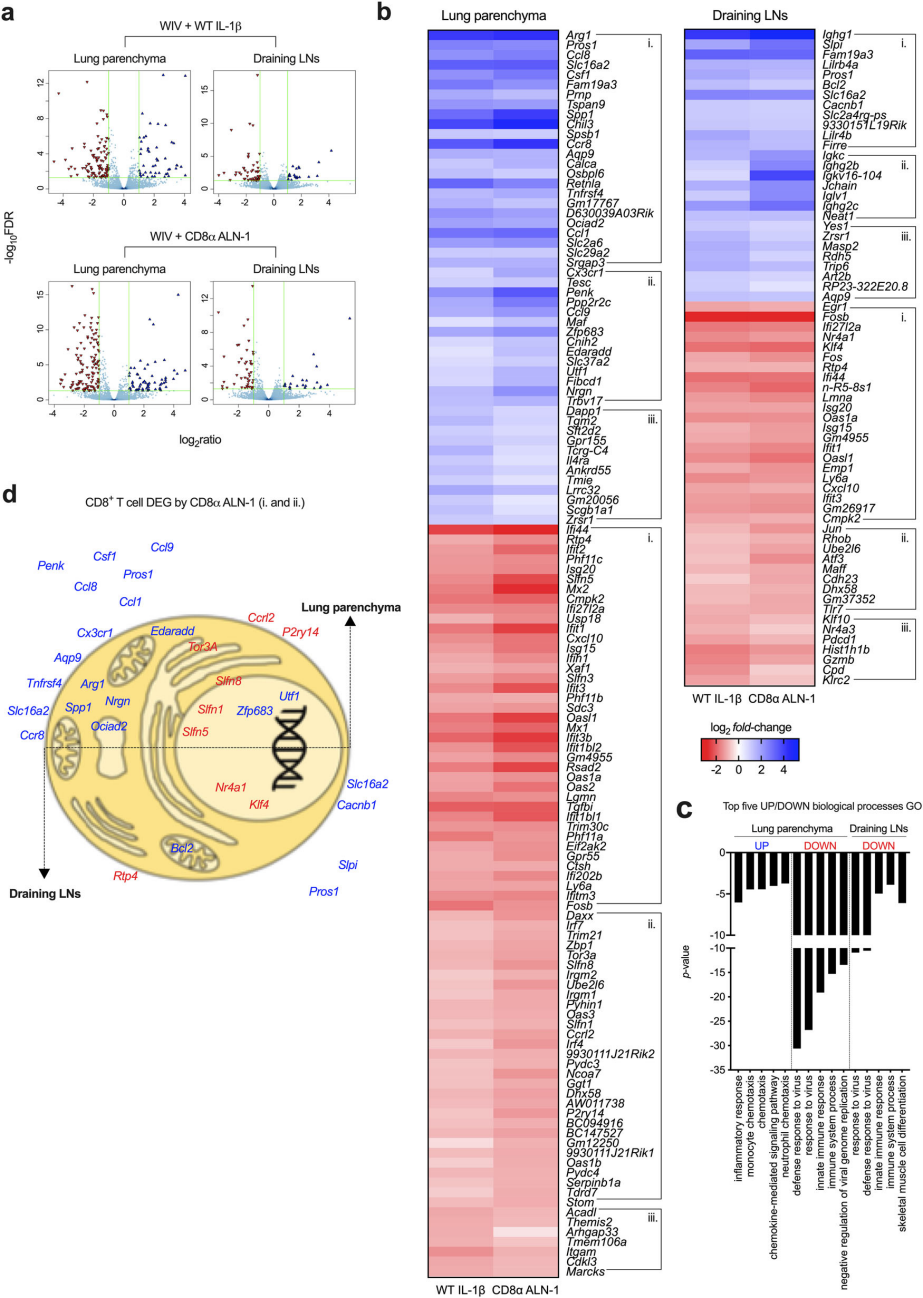
The transcriptional landscape of CD8^+^ T cells isolated from vaccinated mice during influenza virus infection supports the cellular adjuvant effect of CD8α ALN-1. **a** Volcano plots showing all genes found differentially up- (blue) or downregulated (red) in CD8^+^ T cells sorted from lung parenchyma (left) or lung-draining mediastinal LNs (right) of mice vaccinated with WIV and WT IL-1β (up) or CD8α ALN-1 (down) compared with mice vaccinated with WIV alone. Significance is indicated by a False Discovery Rate (*FDR*) < 0.05 and an absolute log_2_ fold change >1. **b** Heat maps of all statistically significant DEGs identified in CD8^+^ T cells isolated from lungs (left map) or draining LNs (right map) of mice vaccinated with WIV and WT IL-1β (left columns) or CD8α ALN-1 (right columns). Cells summarize the log_2_ fold change in the gene expression level of *n* = 3 mice/group, compared with vaccination with WIV alone. Clusters indicate DEGs shared between (i.) or unique for treatment with WT IL-1β (iii.) and/or CD8α ALN-1 (ii.). Within these clusters, genes are organized by increasing *FDR*. **c** Top five of up- and downregulated GO biological processes, clustered using the DAVID bioinformatics tool based on the lists of DEGs identified in lungs and draining LNs. **d** Graphical representation of the subcellular localization of gene products with a known association with regulation of CD8^+^ T cell activation, survival and memory development (based on the literature). See also Supplementary Fig. 8.

The transcriptome of CD8^+^ T cells isolated from mice vaccinated with WIV and WT IL-1β or CD8α ALN-1 was significantly altered in lung (50 upregulated and 76 downregulated genes) and to a lesser extent in draining LNs (27 upregulated and 38 downregulated genes) compared with mice vaccinated with WIV alone (Fig. 7a, b, Supplementary Fig. 8c). For mice vaccinated with WIV and WT IL-1β or CD8α ALN-1, all genes that were found to be significantly up- or downregulated compared with WIV alone vaccinated mice are summarized in Supplementary Table 2.

We summarized the gene ontology (GO) biological processes (BPs) associated with these transcriptome changes by performing DAVID bioinformatics analysis^47^ using the differentially expressed genes (DEGs) in lung or draining LNs as input. Among the top-five most upregulated BPs were the inflammatory response and different chemotactic processes, indicative for immune cell recruitment to the site of viral infection (Fig. 7c). Furthermore, this GO analysis indicated that genes associated with the antiviral response to influenza (e.g., *Ifi27l2a*, *Isg20*, *Oas1a*, *Isg15*, *Ifit1*, *Oasl1*, and *Ifit3*) were strongly enriched in mice vaccinated with WIV alone as compared with mice vaccinated with WT IL-1β or CD8α ALN-1.

We performed a structured literature search to correlate the identified transcriptional changes in CD8^+^ T cells to the enhanced T cell responses and survival rates after viral challenge observed in mice vaccinated with WT IL-1β or CD8α ALN-1. We found that several transcripts with previously reported roles in long-term survival of T cells (e.g., *Cacnb1*, *Bcl2* and *Aqp9* and *Arg1*)^36,48–51^, installation of peripheral residency (e.g., *Zfp683* or *Hobit*)^52^, activity of T cells (e.g., *Pros1* and *Tnfrsf4*)^53,54^ and T cell effector functions (e.g., *Cx3cr1*)^55^ were significantly upregulated in CD8^+^ T cells isolated from mice vaccinated with WT IL-1β and CD8α ALN-1. On the other hand, downregulation of several *Schlafen*-family genes (e.g., *Slfn 1, 3, 5*, and *8*) was found in these cells, which has been described before as a consequence of CTL activation, allowing T cells to exit a quiescent state^56^. We further summarized DEGs with known importance for different CD8^+^ T cell-related processes in Fig. 7d.

Of note, in draining LNs from mice vaccinated with WIV and CD8α ALN-1, we retrieved several genes associated with the immunoglobulin response (e.g., *Igkc*, *Ighg2b*, and *Jchain*), mediated by B lymphocytes. These results are probably due to sample contamination with small amounts of B cells (Fig. 7b).

Altogether, these transcriptomics data indicate that WT IL-1β and CD8α ALN-1 provoke changes in the transcriptome of CD8^+^ T cells that are in line with the generation of potent and active CTLs both in lung parenchyma and draining LNs.

## Discussion

The ability of IL-1β to potentiate T cell responses to antigens has been recognized for several decades^13–17^. Our study further endorses the observations made by Ben-Sasson et al.^16^, as we show that systemic delivery of WT IL-1β to mice enhanced the proliferation and effector functions of CD8^+^ T cells in response to the OVA model antigen compared with mice immunized with antigen alone. However, as expected, this treatment elicited toxicities due to side effects of WT IL-1β, which include severe body weight loss, induction of a cytokine storm, as evidenced by the release of proinflammatory IL-6, and platelet destruction. Systemic toxicities associated with WT IL-1β administration are indeed well known, both in mice and men^25–27^, and have hampered clinical translation. We reasoned that restriction of IL-1β activity to CD8^+^ T lymphocytes could be a solution for safe exploitation of its beneficial properties. Therefore, we coupled a CD8α-binding sdAb to IL-1β Q148G, a single point mutant with strongly (168-fold) reduced biological activity. As such, we created a cytokine that can regain activity upon CD8^+^ T cell targeting (CD8α AcTaleukin/CD8α ALN-1). In different assays, CD8α ALN-1 induced CTL responses to the same extent as WT IL-1β, but unlike WT, treatment of mice with a corresponding dose of CD8α ALN-1 was not associated with toxic side effects causing measurable morbidity. Despite a large reduction in systemic toxicity, CD8α ALN-1 triggered lymphopenia in peripheral blood, comparable to WT IL-1β delivery. This significant decrease in different subsets of circulating lymphocytes occurred within the first 6 h after CD8α ALN-1 delivery and was maintained over multiple treatments. Although we were unable to demonstrate lymphocyte redistribution to the tissues in response to CD8α ALN-1 administration, we hypothesize that the lymphopenia might result from low residual activity of CD8α ALN-1 on endothelial cells, which are known to be extremely sensitive to IL-1β due to their high surface expression of IL-1R1^57^. Alternatively, this could also be a consequence of endogenous IL-1β release by cDC1s that might be targeted and subsequently activated upon CD8α ALN-1 delivery^58^.

As CD8α ALN-1 was found to induce effective T cell responses to antigen with a vastly improved safety profile, we decided to evaluate its protective potential as an adjuvant in a model for prophylactic influenza vaccination. Seasonal influenza vaccines are usually not adjuvanted and they are poor at inducing cellular immune responses as their protective potential relies on the generation of neutralizing antibodies. Nevertheless, safe adjuvants that promote the response of CD8^+^ T cells might find application in universal influenza vaccines, during pandemics (where fast cross-protective responses and antigen dose sparing are required) and to improve protection of high-risk groups that respond poorly to the licensed influenza vaccines (e.g., elderly)^7,59^. Different recent studies indeed argue that strong CD8^+^ T cell responses are pivotal in influenza control^3–6^.

Six adjuvants have been licensed for use in human influenza vaccines: alum; the oil-in-water emulsions MF59, AS03, and AF03 (currently not in use); virosomes (current use is unclear) and heat-labile enterotoxin (withdrawn due to safety concerns and therefore not in use). Most of these adjuvants are safe and predominantly raise effective antibody and T helper 2 responses to coadministered antigens, yet, they are cautiously used in influenza vaccines, in part because of the limited mechanistic knowledge on their mode of action. This lack of knowledge has never been a problem for market approval, but could become problematic when side effects would occur^59^. Therefore, we examined CD8α ALN-1’s mode of action in greater detail. Efficient targeting of IL-1β Q148 to CD8^+^ T lymphocytes and type I cDCs is mediated by a CD8α sdAb with a low-nanomolar K_D_. The activity of CD8α ALN-1 is restored upon target cell binding and the efficiency of this targeting effect varied from complete restoration in HEK-Blue-IL1R reporter gene experiments to partial restoration of NF-κB-, p38 MAPK-, and AP-1-induced gene expression in 132 1N1 astrocytes, which is probably a consequence of lower target expression in 132 1N1 astrocytes after transfection. Indeed, we found that the activity of CD8α ALN-1 upon targeting is strongly dependent on the level of target antigen expression. Considering this, CD8α could be regarded as a strong target candidate due to its quite selective expression pattern and high amount of copies documented per individual CD8^+^ T cell^60^.

Binding of CD8α ALN-1 was found to be completely dependent on the targeting moiety that concentrates the construct on the plasma membrane, as binding was unaltered on target cells that do not express IL-1R1. Targeting of type I cDCs can be explained by their known expression of CD8α, which is unique for mouse. During influenza virus infection, IL-1β signaling in DCs was demonstrated to be both necessary and sufficient for the induction of antiviral CD8^+^ T cell responses^22^. Conversely, during immunization of healthy mice, it was observed that the adjuvant effect of IL-1β only depended on IL-1R1 expression on antigen-specific CD8^+^ T cells^16^. Our work supports this latter finding and shows that in a vaccination setting, the adjuvant properties of CD8α ALN-1 remained intact when IL-1R1 expression was restricted to the OVA-specific CD8^+^ T cells. However, as this experiment only shows untouched proliferation of OT-Is in the absence of peripheral IL-1R1, it cannot fully exclude a role for type I cDCs in the adjuvanticity of CD8α ALN-1. This is important for translation towards a human system, where cross-presenting DCs are known for their CD8α negativity and additional studies, such as OT-I transfer experiments in Batf3^−/−^ C57BL/6 mice, could be performed to resolve this issue^61^.

We demonstrated that inclusion of CD8α ALN-1 in a prime-boost model of prophylactic influenza vaccination with whole-inactivated H3N2 virus protects mice against challenge with a heterosubtypic pH1N1 virus to the same extent as WT IL-1β, whereas most of the mice that had been vaccinated without adjuvant rapidly succumbed to infection-induced disease. As intended, this protection fully correlated with the induction of functionally potent and long-lasting antiviral T cells. One day prior to viral challenge, we found that CD8α ALN-1 promoted both CD8^+^ and CD4^+^ T cell responses against epitopes of influenza’s NP. These CD4^+^ T cell responses could be resulting from *trans*-effects, possible due to the flexible stretch of 60 amino acids separating the mutated cytokine and the sdAb, of CD8α ALN-1 on proximal CD4^+^ helper T cells, as these cells collaborate in close contact during the formation of an antigen-specific CTL response. Earlier work already demonstrated that IL-1β can act directly on CD4^+^ T cells, enhancing their proliferation, activation and differentiation into memory phenotypes upon antigen recognition^17^. However, we cannot exclude the possibility that IL-1β activity on CD8α^+^ type I cDCs might contribute to the antiviral CD4^+^ T cell response. Earlier work demonstrated that CD4^+^ T cells can be activated by type I cDCs through MHC-II antigen presentation^62^ and it has been shown, using Clec9A-DTR mice, that ablation of type I cDCs can lead to diminished CD4^+^ T cell responses^63^. Clec9A targeting on type I cDCs has also been proven to efficiently generate vaccine antibody responses^64^. Our data suggest that the observed protection against influenza after vaccination with CD8α ALN-1 is mediated by equally strong CD4^+^ and CD8^+^ T cell responses, which is in accordance with the recent work of Lapuente et al., who demonstrated that WT IL-1β induces both CD4^+^ and CD8^+^ T_RM_ cells against influenza NP and proved, in FTY720 experiments, that these T_RM_ cells are necessary and sufficient to control viral replication at the mucosal site of infection^24^.

We aimed to study the CD8^+^ T cell response in greater detail by sequencing RNA from CTLs, isolated from lungs and lung-draining mediastinal LNs 1 week after viral infection. We found several interesting transcripts that were enriched in CTLs from mice vaccinated with WT IL-1β and/or CD8α ALN-1. For example, we noticed upregulation of *Arg1* and *Aqp9*, two IL-7-regulated genes that were both shown to be involved in T cell metabolism and found to be essential for the fitness, survival, and longevity of T cells by regulation of l-Arginine metabolization and intracellular glycerol transport, respectively^50,51^. *Tnfrsf4* has been described before to regulate activation of T cells in the context of vaccination^54^ and was found enriched in these CTLs. We also identified several upregulated genes that have a known correlation with memory formation. *Spp1* (or *Osteopontin*) was shown to modulate the generation of memory CD8^+^ T cells by regulation of the cytokine milieu during influenza A virus infection^65^. *Ccr8* transcripts, on the other hand, were found by different groups in T_RM_ cells after the induction of viral infection^66,67^. We also found upregulation of *Ccl1* and *Ccl9* in CD8^+^ T cells isolated from mice vaccinated with WT IL-1β and CD8α ALN-1 and both genes were previously found to be enriched in memory T cells after influenza infection^68^. Among the genes only upregulated upon vaccination with CD8α ALN-1 we found *Edaradd*, which was described earlier in the formation of long-living T_RM_ cells during antiviral responses^69^, and *Cx3cr1*, which is known to be expressed in the predominant subset of memory T cells that surveys peripheral tissues^55^. Work by Mackay et al. has shown that *Zfp683* (or *Hobit*), which was found to be induced after CD8α ALN-1 vaccination only, is a master regulator of a transcriptional program that instructs residency and tissue retention in T cells, steering those towards a T_RM_ phenotype^52^.

These transcriptomics data suggest that CD8^+^ T cells of mice vaccinated with WT IL-1β and CD8α ALN-1 might be superior to their counterparts isolated from WIV only vaccinated mice, which could explain the better antiviral protection observed in these mice. These data also support the phenotypic profiling that we performed using flow cytometry at the same timepoint as the RNA sequencing. In the lung parenchyma, the antiviral CD8^+^ T cells induced upon WT IL-1β and CD8α ALN-1 treatment were primarily CD62L^−^CD44^+^CD69^+^, corresponding to a T_RM_ phenotype, which is in line with the observed expression of *Hobit*, among other genes. Importantly, we could retrieve these antiviral CD8^+^ T cells in the lung parenchyma of mice treated with WT IL-1β or CD8α ALN-1 more than 50 days after the initial infection, illustrating the longevity of these cells. In the lung-draining LNs, these cells were mainly CD62L^+^CD44^+^, corresponding to a T_CM_ phenotype with higher expression of CD127 compared with the T_EM_-cell subset^45^. T_CM_ cells were described to actively upregulate *Bcl2* to prolong survival^70,71^ and we found enrichment of *Bcl2* in CTLs isolated from LNs of WT IL-1β and CD8α ALN-1 vaccinated mice.

RNA sequencing also revealed a clear enrichment of IFN-regulated genes in both lungs and draining LNs of mice immunized with WIV alone, compared with mice vaccinated with WT IL-1β or CD8α ALN-1. The presence of antiviral response genes is indicative for active suppression of viral replication^72^ and therefore this data is indirect evidence that, at this timepoint, only mice vaccinated with WT IL-1β or CD8α ALN-1 had successfully cleared the infection. However, our analysis was limited as it provides only a snapshot of the CTL transcriptome at a selected timepoint after viral infection. Moreover, these gene expression data are no result of direct IL-1β signaling on CTLs as the last treatment was administered three weeks prior to this readout. Future work should therefore focus in greater detail on how IL-1β signaling in CD8^+^ T cells can drive differentiation into these favorable states. Also a more precise dissection of how IL-1β promotes the formation of different memory T cell subsets could be informative for the development of new adjuvants.

In summary, we presented CD8α ALN-1 as a cellular adjuvant that promotes CD8^+^ T cell responses. CD8α ALN-1 uniquely positions itself among other adjuvants, due to its well understood mode of action and safety profile (unlike many other PRR agonists). CD8α ALN-1 governs protection against a high viral inoculum likely by raising functional and long-lasting antiviral T cells. We see further application of this adjuvant in other models that depend on inducing cellular immunity, including cancer, chronic viral infections, and infections with intracellular pathogens.

## Methods

### In silico analyses of the IL-1β/receptor interactions

Effects of mutations on the IL-1β/receptor interactions were predicted using the crystal structure of the human IL-1β with the extracellular fragments of its receptors (PDB code 1DEP)^73^. The average of five calculations with the Fold-X 4.0 command PSSM was used to predict the *ΔΔG* of the IL-1β Q148G mutant on its interaction with the IL-1R1^74^. Buried surface area of the mutated residue and contacts of the mutated residue were derived using PDB-ePISA and by visualization of the crystal structure using UCSF Chimera^75,76^.

### Molecular cloning and recombinant protein production and purification

The Q148G mutation was introduced in the coding sequence of human IL-1β by site-directed mutagenesis (Q5® Hot Start High-Fidelity DNA Polymerase, M0493L, New England BioLabs Inc.) (5′-GAAGGCACTGCATCTGGGTGGCCAGGACATGGAACAGC-3′ (forward); 5′-GCTGTTCCATGTCCTGGCCACCCAGATGCAGTGCCTTC-3′ (reverse complement), primers were synthesized and purified by Eurogentec). Anti-mouse CD8α and BcII10 sdAbs were generated by the VIB Protein Service Facility, as described previously^29^. WT IL-1β, CD8α WT IL-1β, CD8α ALN-1, BcII10 ALN-1, and CD8α hIFNα2 were constructed in an in-house developed vector (pmTW). Plasmid DNA was purified from the supernatant of DH10B *E. coli* bacteria (18290-015, Thermo Fisher Scientific) after overnight growth (37 °C, 175 rpm) by anion exchange chromatography, using the NucleoBond® PC 2000 system (740525, Machery Nagel). Proteins were produced in FreeStyle^TM^ 293-F cells (R79007, Thermo Fisher Scientific), grown in suspension and transfected at a density of 1.2 × 10^6^ cells/mL in 300 mL of FreeStyle^TM^ 293 expression medium (12338026, Thermo Fisher Scientific) using the PEI-25k transfection reagent (600 μg) (23966-2, PolySciences Inc.), complexed with DNA (300 μg). Additional medium (100 mL) was added 72 h post transfection, and 48 h later the supernatant was collected and filtered. Proteins were purified overnight at 4 °C by immobilized metal affinity chromatography, using nickel ion-loaded sepharose resins (17526801, GE Healthcare). Resins were washed with two column volumes of 20 mM imidazole (8.14223.0250, Merck Millipore) in PBS (14190-169, Thermo Fisher Scientific) and proteins were eluted with 400 mM imidazole in PBS. Imidazole was exchanged with PBS by gel filtration using PD-10 columns (17-0851-01, GE Healthcare). Protein concentrations were determined by measuring absorbance at 280 nm. Purity was assessed by SDS-PAGE and Instant Blue (EP ISB1L, Expedeon) staining of the protein gel.

### Cell lines and culture conditions

HEK-Blue-IL1R cells (Invivogen) and 132 1N1 human astrocytes (a gift from Julia Müller, University of Bonn) were cultured in DMEM (41966-052, Thermo Fisher Scientific) supplemented with 10% fetal bovine serum (10270-106, Thermo Fisher Scientific), following conditions specified by the provider. All cells were grown at 37 °C in a humidified atmosphere containing 5% CO_2_ and tested negative for contamination with mycoplasma (Venor^TM^GeM Mycoplasma Detection Kit, PCR-based, MP0025, Sigma-Aldrich). No full authentication of the cell lines was performed.

### In vitro bioactivity and activation-by-targeting proof of concept experiments

For reporter gene experiments, HEK-Blue-IL1R cells were plated 24 h prior to transfection in 75 cm^2^ flasks (1 × 10^6^ cells/flask). Cells were transfected with 1 μg of NF-κB-3kB-Luc reporter gene DNA^77^, using calcium phosphate precipitation. In the activation-by-targeting experiments, this transfection mix was additionally complemented with 10 μg of DNA encoding mouse CD8α or an irrelevant target (pMET7 vector). Transfected cells were plated in 96-black bottom well plates 24 h post transfection (1 × 10^5^ cells/well). Cells were stimulated 24 h later with WT IL-1β, CD8α WT IL-1β or CD8α ALN-1 in a concentration range. Cells were lysed 6 h post stimulation and incubated with luciferase substrate, after which luminescence was measured on an Envision luminometer. NF-κB activity was calculated as fold induction by normalization to the activity in unstimulated cells (bioactivity experiments) or cells stimulated with WT IL-1β (activation-by-targeting experiments).

### Confocal microscopy

For confocal imaging, HEK-Blue-IL1R cells were seeded in six-well plates (2.5 × 10^5^ cells/well). The next day, cells were transfected with either DNA encoding Flag-tagged mouse CD8α or irrelevant target DNA (both 0.5 μg) using lipofectamine-2000 (11668019, Thermo Fisher Scientific). After 24 h, transfected cells were detached using enzyme-free cell dissociation buffer (13151014, Thermo Fisher Scientific), washed and resuspended in DMEM with 10% fetal bovine serum in a 1:1-ratio (50,000 cells/mL). Next, cells (200 µL/well) were transferred to eight-well chambered coverslips (80826, Ibidi), coated with poly-L-lysine (P4707, Sigma-Aldrich). After 24 h, cells were treated for 30 min with vehicle or WT IL-1β, CD8α ALN-1 or untargeted ALN-1 (0.5 nM). At the end of the stimulation, cells were rinsed with PBS, containing Ca^2+^ and Mg^2+^ (14040133, Thermo Fisher Scientific) and fixed for 15 min at room temperature in paraformaldehyde in PBS (4%). After three PBS washes, cells were permeabilized with Triton X-100 in PBS (0.1%) for 10 min and blocked in BSA in PBS (1%) for another 10 min at room temperature. Samples were then incubated overnight (4 °C) with mouse anti-Flag (2000× diluted) (F3165, Sigma) and rabbit anti-NF-κB p65 p-Ser536 (400× diluted) (sc-372, Santa Cruz) in BSA in PBS (1%). After three washes in PBS, cells were incubated for 30 min at room temperature (dark) with anti-rabbit Alexa 568 (500× diluted) (A10042, Thermo Fisher Scientific) and anti-mouse Alexa 488 (A11029, Thermo Fisher Scientific) (500× diluted) in BSA in PBS (1%). DAPI (2 µg/mL) was added to the secondary antibody mix to stain the nuclei. Next, cells were washed four times in PBS and covered with propyl gallate mounting medium. Images were acquired and analyzed using Fluoview 1000 software.

### RNA isolation and RT-qPCR

One hundred and thirty-two 1N1 cells were seeded in six-well plates (2.5 × 10^5^ cells/well). The next day, cells were transfected with 0.5 μg of DNA encoding mouse CD8α or an irrelevant target protein using lipofectamine-2000. After 24 h, transfected cells were detached using trypsin-EDTA (25300096, Thermo Fisher Scientific), washed with PBS and resuspended in DMEM, supplemented with 10% fetal bovine serum (2 × 10^5^ cells/mL). Cells were transferred to 24-well plates (1 × 10^5^ cells/well) and stimulated the next day with vehicle or WT IL-1β, CD8α ALN-1, or untargeted ALN-1 (0.5 nM). After 6 h of treatment, cells were washed with PBS and total RNA was extracted using the RNeasy Mini Kit (74104, Qiagen). Reverse transcription was performed on 0.5 μg of total RNA using the PrimeScript RT Reagent kit (RR037A, Takara). For real-time cDNA amplification we used the LightCycler® 480 SYBR Green I Mastermix (04707516001, Roche) and the primers listed in Supplementary Table 1. Samples were amplified and analyzed using the LightCycler® 480 System (Roche). Relative quantification of mRNA expression was performed using ΔΔCt analysis with HPRT as reference gene.

### Mice

All mice were housed under pathogen-free conditions in individually ventilated cages, placed in a temperature-controlled environment with a 12 h day/12 h night cycle. Mice received food and water ad libitum. Female C57BL/6 and BALB/c mice were purchased from Charles River Laboratories. OT-I T cell receptor (TCR) transgenic CD45.1 C57BL/6 RAG2^−/−^ mice (a gift from Filipe Branco Madeira) and C57BL/6 IL-1R1^−/−^ mice (a gift from Andy Wullaert, both from the VIB-UGent Center for Inflammation Research) were bred at our own facility. All mice were between 7 and 12 weeks of age at the start of experiments. All animal experiments followed the guidelines of the Federation of European Laboratory Animal Science Association and were approved by Ethical Committees of Ghent University (ECD 17/80k, ECD 18/127k, ECD 16/07, and ECD 2018/86). For all experiments, mice were allocated to groups randomly. The investigators were not blinded during data collection nor analysis.

### Binding studies on murine splenocytes in vitro

Spleens were isolated from C57BL/6 or C57BL/6 IL-1R1^−/−^ mice, collected in PBS and passed through 70 μm nylon strainers by mashing. Red blood cells were lysed with a home-made lysis buffer (155 mM Na_4_Cl, 12 mM NaHCO_3_, and 127 μM EDTA in PBS pH 7.4). Cells were resuspended in home-made FACS buffer (1% FBS, 0.09% sodium azide and 0.05 mM EDTA in PBS), plated in a 96-well plate (1 × 10^6^ cells/well) and incubated for 2 h at 4 °C with WT IL-1β, CD8α ALN-1, or BcII10 ALN-1 (10 nM for type I cDC binding, 1 nM for binding on all other cell subsets, concentration range for titration experiments). Cells were stained in FACS buffer with anti-CD16/32 (100× dilution) (Thermo Fisher Scientific) to block Fc receptors, followed by staining with LIVE/DEAD Fixable Aqua (1000× dilution) (Thermo Fisher Scientific), CD19 AF700 (500× dilution) (clone eBio1D3, 56-0193-82, Thermo Fisher Scientific), CD3 AF700 (clone 17A2, 56-0032-82, Thermo Fisher Scientific) or PE-Cy7 (250× dilution) (clone 145-2C11, 552774, BD Biosciences), CD4 PE (250× dilution) (clone RM4-5, 553048, BD Biosciences), CD11b PE-Cy7 (250× dilution) (clone M1/70, 101215, BioLegend), CD11c APC (100× dilution) (clone N418, 117310, BioLegend), XCR1 PE (100× dilution) (clone ZET, 148204, BioLegend), and His-tag FITC (2000× dilution) (clone 6G2A9, A01620, GenScript). Cells were recorded on a four-laser Attune Nxt flow cytometer (Thermo Fisher Scientific) and data were analyzed using FlowJo software (Treestar). First, we selected single cells based on FSC/SSC and living cells based on negativity for LIVE/DEAD. Within this subset, we described CD19^+^ cells as B cells, CD19^−^/CD3^+^/CD4^+^ cells as CD4^+^ T cells, CD19^−^/CD3^+^/CD4^−^ cells as CD4^−^ T cells (corresponding to CTLs), CD19^−^/CD3^−^/CD11c^+^ as cDCs, CD19^−^/CD3^−^/CD11c^+^/XCR1^+^ cells as type I cDCs, and CD19^−^/CD3^−^/CD11b^+^ cells as myeloid cells.

### In vitro OT-I coculture assay

Bone marrow was isolated from tibias and femurs of C57BL/6 mice by flushing the bones with PBS. Red blood cells were lysed as described above and cells were seeded in six-well plates (1 × 10^5^ cells/mL) in RPMI-1640 (61870-044, Thermo Fisher Scientific), supplemented with 5% Fetal Clone I, recombinant mouse GM-CSF (20 ng/mL) (both a gift from Karine Breckpot, Vrije Universiteit Brussel), gentamicin (100 μg/mL) (15710-049, Thermo Fisher Scientific), and 50 μM β-mercaptoethanol (21985-023, Thermo Fisher Scientific). After 10 days of culture, mature BM-DCs were plated in six-wells (2.5 × 10^6^ cells/mL) and pulsed with 10, 100 pM or 1 nM SIINFEKL (OVA_257–264_) (AS-60193-1, AnaSpec) for 1 h at 37 °C, 5% CO_2_. Spleens were isolated from OT-I TCR transgenic CD45.1 C57BL/6 RAG2^−/−^ mice and processed to single cells as described before. CD8^+^ T cells were purified by negative selection using magnetic-activated cell sorting (MACS) (130-104-075, Miltenyi Biotec), following the manufacturer’s instructions. These purified OT-I cells express a transgenic TCR, recognizing SIINFEKL. Cells were subsequently labeled with 5 μM carboxyfluorescein succinimidyl ester (CFSE) (65-0850-84, Thermo Fisher Scientific) and plated (1 × 10^5^ cells/well) together with the loaded BM-DCs (1 × 10^4^ cells/well) in 96-well plates for 72 h, supplemented with inhibitory antibody (2000× dilution) (clone CT-CD8a, MA5-17594, Thermo Fisher Scientific), WT IL-1β or CD8α ALN-1 (1 nM). Following this coculture, cells were stained in FACS buffer with anti-CD16/32, CD3 PE-Cy7, CD4 PE, and CD25 APC (100× dilution) (clone PC61.5, 17-0251-81, Thermo Fisher Scientific). Samples were recorded on the Attune Nxt flow cytometer and data were analyzed using FlowJo software. First, we selected single cells based on FSC/SSC. Within this subset, we described OT-I cells as CD3^+^/CD4^−^/CFSE^labeled^.

### In vivo OT-I proliferation experiments

CD8^+^ OT-I cells were isolated and MACS-purified as described above. Purified cells were labeled with 5 μM CTV (C34557, Thermo Fisher Scientific), according to the manufacturer’s instructions, and i.v. adoptively transferred in C57BL/6 recipient mice (1.5 × 10^6^ cells/mouse in 200 μL PBS). One day post transfer, recipient mice were immunized i.p. with endotoxin-free full-length OVA protein (100 μg/mouse in 50 μL PBS) (vac-pova-100, Invivogen). Starting together with the antigen delivery, mice received additional i.p. treatments with LPS (25 μg/mouse) (tlrl-eklps, Invivogen), WT IL-1β (5 μg/mouse), CD8α ALN-1 (10 μg/mouse, which is an equimolar dose to WT IL-1β), or BcII10 ALN-1 (10 μg/mouse, all in 100 μL PBS) every 24 h for 3 consecutive days. One day after the last adjuvant treatment, spleens were isolated and processed to single cells as described above and stained in FACS buffer with anti-CD16/32, CD19 AF700, CD3 PE-Cy7, CD4 PE, CD45.1 BV605 (100× dilution) (clone A20, 110737, BioLegend), SIINFEKL in H2-K^b^ pentamer APC (10 μL/sample, following the manufacturer’s instructions) (F093-4A, ProImmune), CD44 PerCP-Cy5.5 (100× dilution) (clone IM7, 103032, BioLegend), and CD62L APC-Cy7 (100× dilution) (clone MEL-14, 104428, BioLegend). Cells were recorded on the Attune Nxt flow cytometer and data were analyzed using FlowJo software. Single cells were selected based on FSC/SSC. OT-I cells were described as CD19^−^/CD3^+^/CD4^−^/CD45.1^+^/SIINFEKL in H2-K^b^ pentamer^+^/CTV^labeled^.

### In vivo killing experiments

Splenocytes were isolated from C57BL/6 mice, processed to single cells as described earlier and suspended in RPMI-1640, supplemented with 10% fetal bovine serum. Half of these splenocytes were pulsed with SIINFEKL (10 μg/mL) for 2 h at 37 °C, 5% CO_2_, while the remaining cells were left unloaded. Peptide-loaded cells were labeled with 5 μM CTV, according to the manufacturer’s instructions, while unloaded cells were labeled with a tenfold lower concentration of CTV (500 nM). Both splenocyte pools were subsequently mixed in a 1:1-ratio and i.v. transferred (1 × 10^7^ cells/mouse in 200 μL PBS) in recipient mice, 5 days after the last adjuvant treatment (the immunization strategy applied is presented earlier for the in vivo OT-I proliferation experiments). One day post transfer, splenocytes were isolated from recipient mice, processed to single-cell suspensions and recorded on the Attune Nxt flow cytometer. Transferred cells were identified as CTV^labeled^ cells after single-cell selection using FSC/SSC. Data were analyzed using FlowJo software, allowing to calculate the percentage of antigen-specific killing as: 100−[100 × (%CTV^high^ treated mice/%CTV^low^ treated mice)/(%CTV^high^ PBS-treated mice/%CTV^low^ PBS-treated mice)]. Prior to transfer, correct CTV labeling and peptide presentation was verified using flow cytometry, by staining the splenocyte pools in FACS buffer with a SIINFEKL in H2-K^b^ PE antibody (100× dilution) (clone 25-D1.16, 141603, BioLegend).

### Hematological analyses

Six hours after the initial delivery of OVA and adjuvant, blood was sampled from the tail vein of treated mice, collected in EDTA-coated microcuvette tubes (Sarstedt), and analyzed in a Hemavet 950FS whole blood counter (Drew Scientific). For the analysis of IL-6 in plasma, blood was centrifuged at 14,000 × *g* for 10 min at 4 °C, after which the cytokine concentration was determined by ELISA (431302, BioLegend), according to the manufacturer’s instruction.

### Influenza viruses and viral infection procedures

X47 (H3N2) and pH1N1 (A/Belgium/145-MA/2009, a mouse-adapted virus derived from a clinical isolate of the H1N1 2009 pandemic virus) were grown on Madin–Darby canine kidney cells in serum-free RPMI-1640, complemented with l-1-tosylamide-2-phenylethyl chloromethyl ketone-treated trypsin (T1426, Sigma-Aldrich). WIV was prepared as described earlier^42^. BALB/c mice were primed by an i.m. injection of WIV (15 μg/mouse in 50 μL PBS), followed by an i.v. injection with WT IL-1β (5 μg/mouse), CD8α ALN-1 (10 μg/mouse), BcII10 ALN-1 (10 μg/mouse), or CD8α hIFNα2 (10 μg/mouse, all in 200 μL PBS) 24 h post WIV delivery. SAS adjuvant (15 μg/mouse) (S6322-1VL, Sigma-Aldrich) was i.m. coadministered with WIV. An identical boost treatment was administered 2 weeks after priming. Two weeks post boost, mice were challenged i.n. under mild isoflurane anesthesia (Abbott Animal Health) with 2 × LD_50_ of the pH1N1 in 50 μL PBS. The body weight of the mice was determined daily, during 14 days, after infection and mice that had lost 25% or more of their initial body weight were euthanized. All influenza virus infections were conducted in a biosafety level 2+ accredited animal facility.

### IFN-γ enzyme-linked immunospot (ELISPOT) assays

For the OVA-specific IFN-γ ELISPOT assays, spleens were isolated from C57BL/6 mice 7 days after the initial OVA immunization and first adjuvant treatment. For the NP-specific IFN-γ ELISPOT assays, spleens were isolated from BALB/c mice 2 weeks after the boost treatment. Splenocytes were processed to single-cell suspensions as described above and seeded (2.5 × 10^5^ cells/well) in a 96-well plate, precoated with an anti-IFN-γ antibody (CT317-T2, U-CyTech biosciences), in the presence of peptide (5 μg/mL) for 24 h. The ELISPOT was further developed according to the manufacturer’s instructions. The peptides used for restimulation of the cells were the MHC-I epitopes OVA_257–264_ (SIINFEKL) and NP_147–155_ (TYQRTRALV) and the MHC-II epitopes NP_206–229_ (FWRGENGRKTRSAYERMCNILKGK), NP_55–77_ (RLIQNSLTIERMVLSAFDERNK) and NP_182–205_ (AVKGVGTMVMELIRMIKRGINDRN) (NP peptides were synthesized and purified by GenScript).

### Analyses of T cell status in lungs and draining lymph nodes upon influenza infection

One week post pH1N1 inoculum, mice were euthanized by overdose with ketamine (80 mg/kg) (Eurovet) and xylazine (5 mg/kg) (Bayer) in 500 µL PBS (i.p.) and perfused with PBS, supplemented with heparin (50 IU/mL) (H5515-100KU, Sigma-Aldrich). Lungs and lung-draining mediastinal LNs were isolated and collected in ice-cold PBS, supplemented with DnaseI (5 IU/mL) (4536282001, Sigma-Aldrich) and liberase (50 μg/mL) (5401119001, Sigma-Aldrich). Lungs were chopped finely using scissors and further minced mechanically using GentleMACS (Miltenyi Biotec). The obtained cell suspension was incubated for 30 min at 4 °C (while rotating) and subjected to another round of GentleMACS mincing. Red blood cells were lysed as described before and finally cells were passed through 70 μm nylon strainers. LNs were mashed, passed through 70 μm nylon strainers and incubated with DNaseI (5 IU/mL) and liberase (50 μg/mL) in PBS for 30 min at 4 °C (while rotating). Single cells isolated from lung and LNs were stained in FACS buffer with anti-CD16/32, LIVE/DEAD Fixable Aqua, CD45 APC-Cy7 (500× dilution) (clone 30-F11, 103116, BioLegend), CD3 PE-Cy7, CD4 BV605 (250× dilution) (clone RM4-5, 100547, BioLegend), CD8 PerCP-Cy5.5 (250× dilution) (clone 53-6.7, 100733, BioLegend), TYQRTRALV in H2-K^b^ pentamer APC (10 μL/sample, following the manufacturer’s instructions) (F098-4A, ProImmune), CD44 BV711 (100× dilution) (clone IM7, 103057, BioLegend), CD62L PE (100× dilution) (clone MEL-14, 104407, BioLegend), and CD127 BV421 (100× dilution) (clone A7R34, 135023, BioLegend) (cells from lung only) or CD69 BV421 (100× dilution) (clone H1.2F3, 104527, BioLegend) (cells from LNs only). Cells were recorded on a five-laser FACSymphony (BD Biosciences) and data were analyzed using FlowJo software. First, we selected single cells based on FSC/SSC and living cells based on negativity for LIVE/DEAD. Within this subset, we identified antiviral CTLs as CD45^+^/CD3^+^/CD4^−^/CD8^+^/TYQRTRALV in H2-K^b^ pentamer^+^ cells. In LNs, T_CM_ cells were further gated as CD44^+^/CD62L^+^ and T_EM_ cells as CD44^+^/CD62L^−^. In lung parenchyma, T_RM_ cells were further gated as CD44^+^/CD62L^−^/CD69^+^ and T_EM_ cells as CD44^+^/CD62L^−^/CD69^−^. Phenotypes were defined as reviewed in^45^.

### CD8^+^ T cell sorting and RNA extraction

Lungs and lung-draining mediastinal LNs were isolated from mice 1 week post pH1N1 infection and processed to single-cell suspensions as described above. Single cells were stained in FACS buffer with anti-CD16/32, LIVE/DEAD Fixable Aqua, CD45 eFluor450 (500× dilution) (clone 30-F11, 48-0451-82, Thermo Fisher Scientific), CD3 PE-Cy7, CD4 FITC (250× dilution) (clone RM4-5, 100510, BioLegend), and CD8 PE (500× dilution) (clone 53-6.7, 12-0081-82, Thermo Fisher Scientific). Samples were run on a three-laser FACSMelody (BD Biosciences) and CD8^+^ T cells were sorted out (4 °C) of the mixed cell populations and captured in 350 μL of RLT Plus lysis buffer, supplemented with β-mercaptoethanol (4 °C) (RNeasy Plus Micro Kit, 74034, Qiagen). First, we selected single cells based on FSC/SSC and living cells based on negativity for LIVE/DEAD. We then identified CTLs as CD45^+^/CD3^+^/CD4^−^/CD8^+^ cells. RNA was isolated following the manufacturer’s instructions (RNeasy Plus Micro Kit, 74034, Qiagen). The concentration, quality and integrity of RNA was assessed using an Agilent 2100 Bioanalyzer (Agilent) and only samples with an RNA integrity number ≥ 8, 280/260 and 260/230 values > 1.8 were sent for sequencing.

### RNA sequencing, data processing, analysis, and statistical identification of DEGs

The VIB Nucleomics Core performed the library preparation and library pooling, two runs of sequencing using an Illumina NextSeq 500 device, processing, and analysis of the sequencing data and the statistical identification of DEGs. For every sample, gene expression levels were first computed. Briefly, the number of reads in the alignments overlapping with gene features were counted for each individual run using the featureCounts R package^78^. These raw count data of both runs were summed, resulting in total merged counts, which were coupled with reference gene annotations. Absent genes, for which all samples had <1 counts per million, were filtered out. Every sample was corrected for its intrinsic GC-content using full quantile normalization on bins of GC-content. The variation in library size and RNA composition between samples was corrected by full quantile normalization using the EDASeq R package^79^. For each sample the normalized gene counts were divided by the total number of counts (in millions) and for every gene, these scaled counts were divided by the gene length (in kbp), resulting in the number of fragments per kilobase of gene sequence and per million fragments of library size (FPKM values). Statistical identification of DEGs was done by statistical modeling of the data, specifying the design of the experiment as follows: log(count) = WIV alone (LN) × β_1_ + WIV alone (lung) × β_2_ + WT IL-1β (LN) × β_3_ + WT IL-1β (lung) × β_4_ + CD8α ALN-1 (LN) × β_5_ + CD8α ALN-1 (lung) × β_6_. The edgeR R package, version 3.20.9, was used to estimate all coefficients β for every gene by fitting a negative binomial generalized linear model (GLM)^80^, using offsets instead of normalized counts. These model estimates allowed for computing the contrasts of interest, here being (1) the WT IL-1β effect in lungs and LNs (WIV alone vs. WIV + WT IL-1β) and (2) the CD8α ALN-1 effect in lungs and LNs (WIV alone vs. CD8α ALN-1). Using edgeR 3.20.9, differential expression (a significant deviation of these contrasts from 0) was tested with a GLM likelihood ratio test. Resulting *p* values were corrected for multiple testing with Benjamini–Hochberg to control the *FDR*. Genes with an *FDR* < 0.05 were selected and considered differentially expressed when the absolute log2 ratio > 1. For every identified DEG, the biological relevance was accounted by checking if the raw counts > 50. We conducted an extensive literature research to select for DEGs with known roles during T cell homeostasis, activation, metabolism, exhaustion, and memory formation (the search consisted of “gene name” AND “CD8 T cell” OR “influenza”).

### Statistical analyses and data presentation

Statistical analyses were performed using the GraphPad Prism 8 software (GraphPad Software). Unless stated otherwise, data are presented as mean ± s.e.m. in all experiments. The number of independent biological replicates or the number of individual mice is shown as “*n*.” Normality of the data was accounted by Shapiro–Wilk testing at the α = 0.05 significance level for every group in the statistical comparison. Normally distributed data sets were compared by unpaired Student’s *t* testing (two-tailed) for the statistical analysis of differences between two groups or ANOVA (one- or two-way or repeated measurements) with Tukey’s or Sidak’s multiple comparisons test for the statistical analysis of differences between more than two groups. Non-normally distributed data were compared by unpaired Mann–Whitney *U* testing (two-tailed) for the statistical analysis of differences between two groups or Kruskal–Wallis testing with Dunn’s multiple comparisons test for the statistical analysis of differences between more than two groups. Log-rank testing was used for the statistical analysis of differences between Kaplan–Meier survival curves. Statistical significance was throughout defined as *p* < 0.05.

### Reporting summary

Further information on research design is available in the Nature Research Reporting Summary linked to this article.

## Supplementary information

Supplementary information

Reporting Summary

## Data Availability

All data supporting the findings of this study are available within the article. All RNA sequencing data are made publicly available by deposition in the Gene Expression Omnibus public database and can be retrieved using the accession number GSE134647 or via the link https://www.ncbi.nlm.nih.gov/geo/query/acc.cgi?acc=GSE134647.
