# Supplementary material for: Specific targeting of IL-1β activity to CD8+ T cells allows for safe use as a vaccine adjuvant
Source: NPJ Vaccines. 2020 Jul 23;5:64. doi: 10.1038/s41541-020-00211-5 (PMC7378068; doi:10.1038/s41541-020-00211-5)
Supplement: Supplementary file 1 — Supplementary information [file 41541_2020_211_MOESM1_ESM.pdf]

Specific targeting of IL-1β activity to CD8$^+$ T cells allows for safe use as a vaccine adjuvant

Bram Van Den Eeckhout[1,2], Lien Van Hoecke[1,3], Elianne Burg[1,2], Sandra Van Lint[1,2], Frank Peelman[1,2], Niko Kley[4], Gilles Uzé[5], Xavier Saelens[1,3,6], Jan Tavernier[1,2,4,*,±] and Sarah Gerlo[1,2,*,±]

[1] VIB-UGent Center for Medical Biotechnology, VIB, 9052 Ghent, Belgium

[2] Department of Biomolecular Medicine, Ghent University, 9000 Ghent, Belgium

[3] Department of Biomedical Molecular Biology, Ghent University, 9052 Ghent, Belgium

[4] Orionis Biosciences Inc., Waltham, 02451 MA, US

[5] CNRS 5235, University of Montpellier, 34090 Montpellier, France

[6] Department of Biochemistry and Microbiology, Ghent University, 9000 Ghent, Belgium

[*] Shared senior authors

[±] Corresponding authors:

VIB-UGent Center for Medical Biotechnology, Albert Baertsoenkaai 3, 9000 Ghent, Belgium

jan.tavernier@vib-ugent.be; sarah.gerlo@ugent.be

SUPPLEMENTARY FIGURES AND LEGENDS 1 – 8

SUPPLEMENTARY TABLES AND LEGENDS 1 – 2

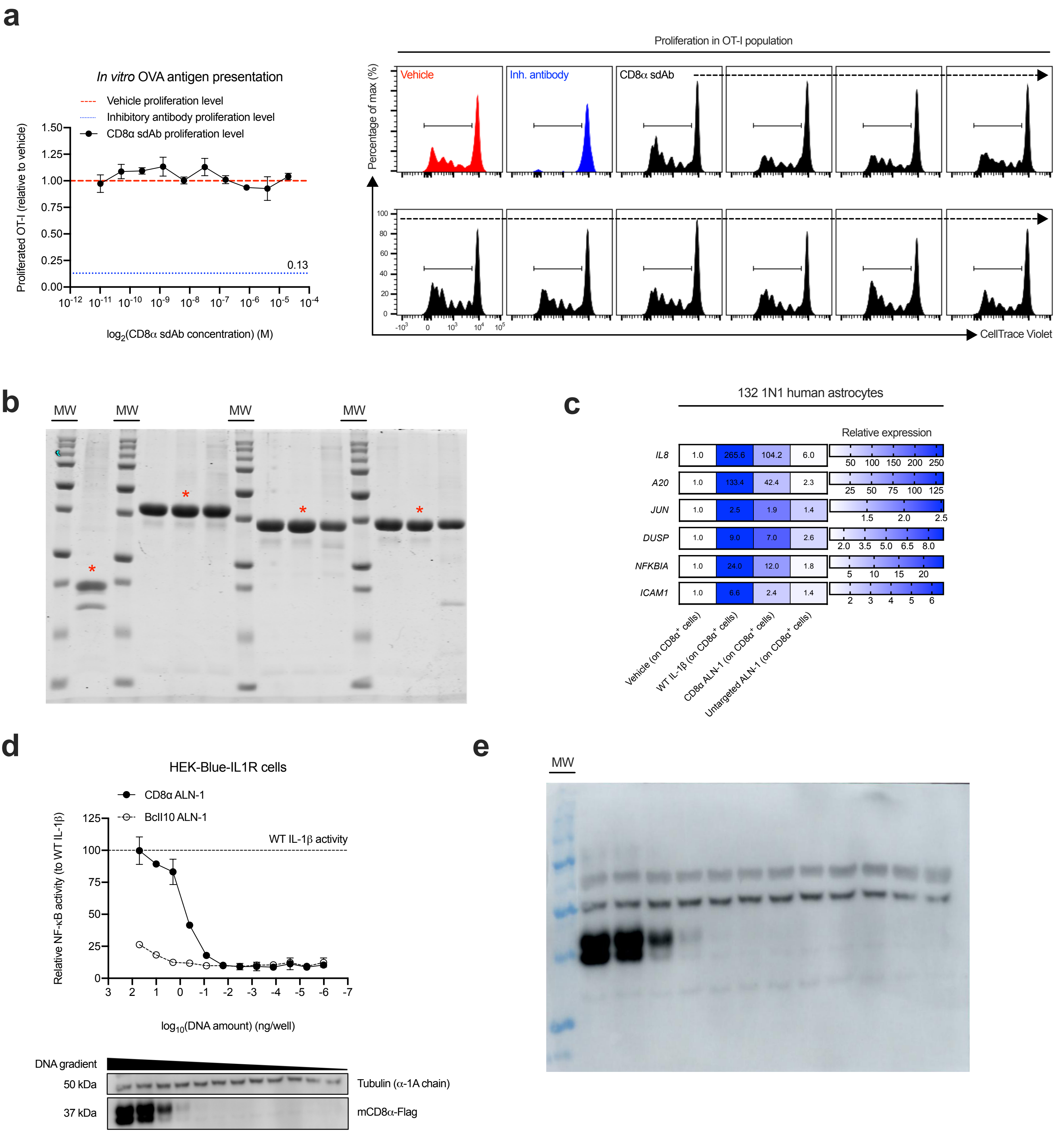

a
In vitro OVA antigen presentation
Vehicle proliferation level
Inhibitory antibody proliferation level
CD8α sdAb proliferation level
Proliferated OT-I (relative to vehicle)
0.13
log2(CD8α sdAb concentration) (M)
Proliferation in OT-I population
Vehicle
Inh. antibody
CD8α sdAb
Percentage of max (%)
CellTrace Violet
b
MW
c
132 1N1 human astrocytes
Relative expression
IL8
A20
JUN
DUSP
NFKBIA
ICAM1
Vehicle (on CD8α+ cells)
WT IL-1β (on CD8α+ cells)
CD8α ALN-1 (on CD8α+ cells)
Untargeted ALN-1 (on CD8α+ cells)
d
HEK-Blue-IL1R cells
CD8α ALN-1
BcII10 ALN-1
WT IL-1β activity
Relative NF-κB activity (to WT IL-1β)
log10(DNA amount) (ng/well)
DNA gradient
50 kDa
37 kDa
Tubulin (α-1A chain)
mCD8α-Flag
e
MW

Supplementary Figure 1. a. The CD8α sdAb does not interfere with $CD8^+$ T cell activation *in vitro*. The CD8α sdAb was tested (20 μM top concentration and 1:5 serial dilution) *in vitro* using the OT-I co-culture system. OT-I activation was assessed by flow cytometry after 72h of culture by evaluating proliferation (CellTrace Violet (CTV) dilution). OT-I cells were detected in the single cell population (based on FSC/SSC) as $CD3^+CD4^-CTV^{labeled}$ cells. *Left:* Quantification of OT-I proliferation. Data points represent the mean proliferation under treatment conditions relative to vehicle ± s.e.m. of one experiment. Treatment with inhibitory antibody is shown as control. *Right:* Representative histograms illustrating unaltered OT-I proliferation in the presence of the CD8α sdAb. **b.** Full and un-cropped image of the representative SDS-PAGE protein gel, with red stars indicating the bands shown in Fig. 1d. **c.** Heat maps showing gene expression (relative to vehicle) of *IL8*, *A20*, *JUN*, *DUSP*, *NFKBIA* and *ICAM1* in $CD8\alpha^+$ 132 1N1 human astrocytes upon stimulation with WT IL-1β, CD8α ALN-1 or untargeted ALN-1 (0.5 nM). Representative heat maps of two independent experiments. **d.** The biological activity of CD8α ALN-1 upon targeting is dependent on the level of target antigen expression. NF-κB-driven luciferase reporter gene expression induced by CD8α ALN-1 (1 nM) in HEK-Blue-IL1R cells, transiently transfected with CD8α (50 ng top concentration and 1:5 serial dilution). *Top:* NF-κB activity is normalized to β-galactosidase activity and expressed as fold induction compared to the activity of WT IL-1β (1 nM) for every tested DNA concentration. Data points represent the mean of two independent experiments ± s.e.m. Untargeted BcII10 ALN-1 (1 nM) is included as control. *Bottom:* Western Blot image of total HEK-Blue-IL1R cell lysates, demonstrating decreasing CD8α protein (37 kDa) levels with decreasing levels of transfected plasmid DNA. Tubulin (50 kDa) is included as loading control. Staining with primary antibodies against Flag-tag (Sigma F7425) and tubulin (Sigma T6199). Secondary detection with HRP-conjugated antibodies against rabbit and mouse IgG (GE Healthcare). Detection by chemiluminescence using the Amersham Imager 680 (GE Healthcare). Representative Western Blot of two independent experiments. **e.** Full and un-cropped image of the representative Western Blot shown in Supplementary Fig. 1d, *bottom*. Samples shown in all gels and blots are derived from the same experiment and were processed in parallel.

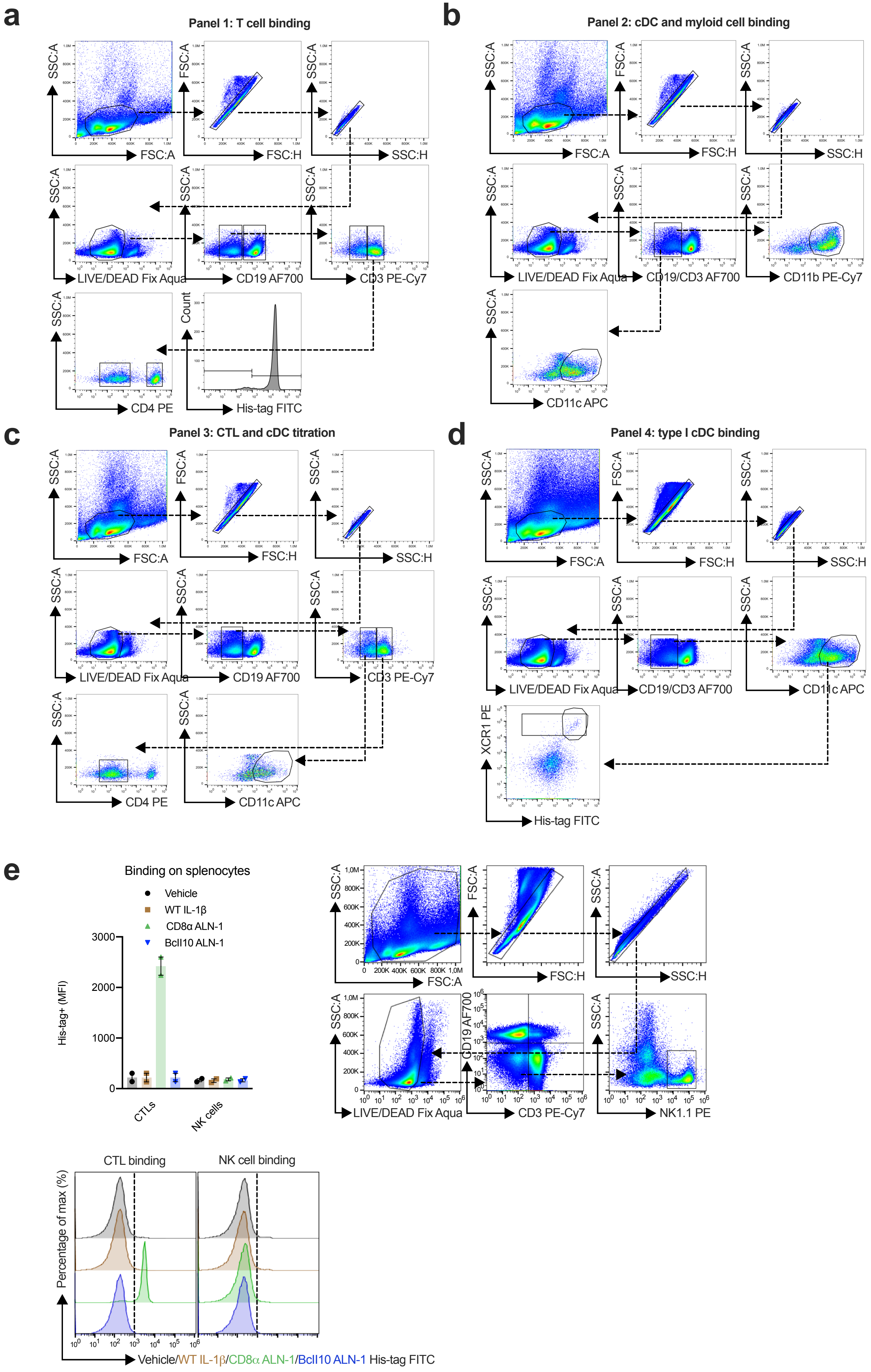
a
Panel 1: T cell binding
SSC:A
FSC:A
FSC:H
SSC:H
LIVE/DEAD Fix Aqua
CD19 AF700
CD3 PE-Cy7
CD4 PE
Count
His-tag FITC
b
Panel 2: cDC and myloid cell binding
CD19/CD3 AF700
CD11b PE-Cy7
CD11c APC
c
Panel 3: CTL and cDC titration
d
Panel 4: type I cDC binding
XCR1 PE
e
Binding on splenocytes
Vehicle
WT IL-1β
CD8α ALN-1
BclI10 ALN-1
His-tag+ (MFI)
3000
2000
1000
0
CTLs
NK cells
NK1.1 PE
CTL binding
NK cell binding
Percentage of max (%)
Vehicle/WT IL-1β/CD8α ALN-1/BclI10 ALN-1 His-tag FITC

Supplementary Figure 2. a – d. Gating strategy for the detection of CD8α ALN-1 binding in C57BL/6 (IL-1R1$^{-/-}$) splenocyte pools via flow cytometry. Staining with LIVE/DEAD dye and CD19, CD3, CD4, CD11b, CD11c, XCR1 and anti-His-tag antibodies. **e.** *Left:* CD8α ALN-1 (1 nM) does not bind NK cells in a mixed pool of C57BL/6 splenocytes, whereas strong CTL targeting is apparent. Bars represent the mean ± s.e.m. of two independent experiments. Controls include vehicle, WT IL-1β and untargeted BcII10 ALN-1 (1 nM) binding. Representative histograms are included below the graph. *Right:* Flow cytometry gating strategy for this analysis. Staining with LIVE/DEAD dye and CD19, CD3, CD4, NK1.1 (100x dilution) (clone PK136, 108707, BioLegend) and anti-His-tag antibodies. NK cells were identified as CD19^{-}CD3^{-}NK1.1^{+} within the single (based on FSC/SSC) and living (LIVE/DEAD^{-}) cell population.

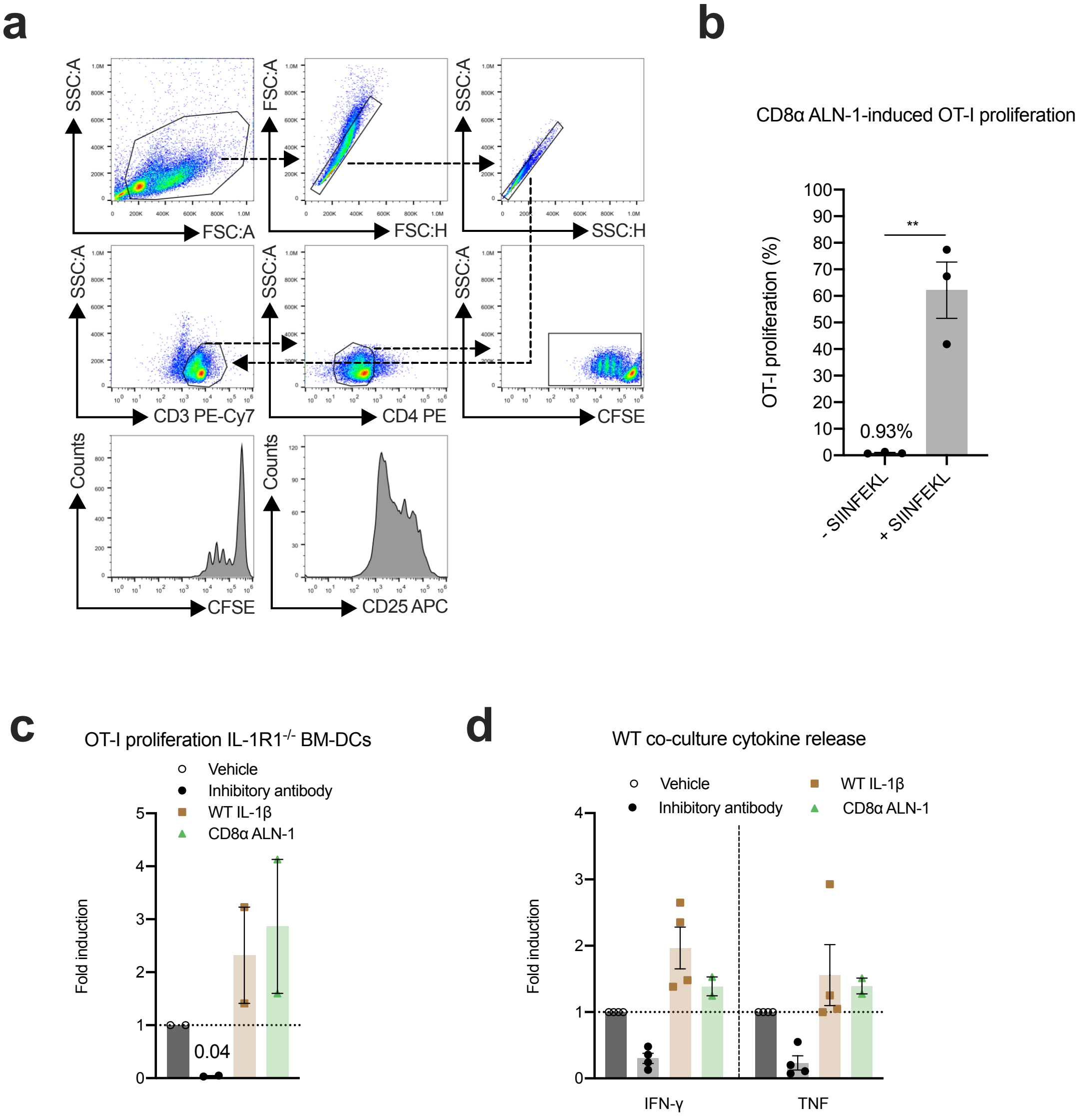
a
SSC:A
FSC:A
FSC:H
SSC:H
CD3 PE-Cy7
CD4 PE
CFSE
Counts
CD25 APC
b
CD8α ALN-1-induced OT-I proliferation
OT-I proliferation (%)
**
0.93%
- SIINFEKL
+ SIINFEKL
c
OT-I proliferation IL-1R1-/- BM-DCs
Vehicle
Inhibitory antibody
WT IL-1β
CD8α ALN-1
Fold induction
0.04
d
WT co-culture cytokine release
Vehicle
Inhibitory antibody
WT IL-1β
CD8α ALN-1
Fold induction
IFN-γ
TNF

Supplementary Figure 3. a. Gating strategy for the *in vitro* OT-I co-culture experiments. Staining with CD3, CD4 and CD25 antibodies and detection of OT-I cells in the single (based on FSC/SSC) cell population as $CD3^{+}CD4^{-}CFSE^{labeled}$ cells. Proliferation is calculated as a measure of CFSE dilution. CD25 upregulation was evaluated in the proliferated OT-I subset. **b.** OT-I proliferation induced upon stimulation with CD8α ALN-1 (1 nM) in the absence or presence of SIINFEKL on BM-DCs. Bars represent the mean ± s.e.m of three independent experiments. **, $p < 0.01$ by unpaired Student's *t*-test (two-tailed). **c.** Flow cytometry analysis of OT-I proliferation (CFSE dilution) upon stimulation with CD8α ALN-1 (1 nM) in *in vitro* OT-I co-cultures with IL-1R1$^{-/-}$ BM-DCs. Controls include the activity of vehicle, inhibitory antibody and WT IL-1β (1 nM). Each bar represents the mean fold induction (CFSE dilution under treatment conditions relative to vehicle) ± s.e.m. of two independent experiments. **d.** ELISA detection of IFN-γ (*left*) and TNF (*right*), released in the conditioned supernatant of *in vitro* OT-I cocultures (with WT BM-DCs) upon stimulation with CD8α ALN-1 (1 nM). Each bar represents the mean fold induction (cytokine release under treatment conditions relative to vehicle) ± s.e.m. of at least two independent experiments. Controls include vehicle, inhibitory antibody and WT IL-1β (1 nM). IFN-γ (CMC4033, Thermo Fisher Scientific) and TNF (DY410, R&D Systems) were detected in the conditioned supernatant by ELISA, following the manufacturer's instructions.

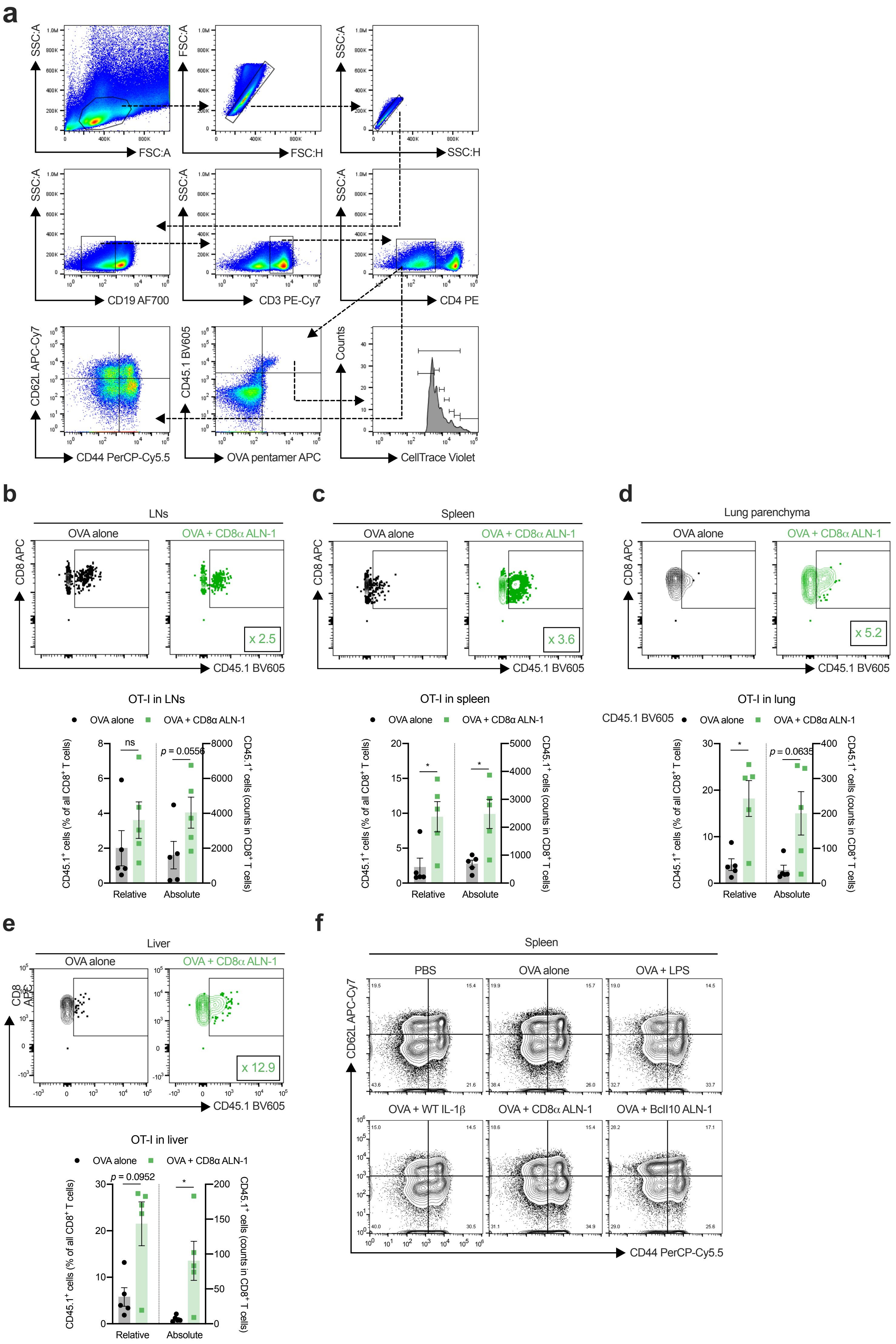
a
SSC:A
FSC:A
FSC:H
SSC:H
CD19 AF700
CD3 PE-Cy7
CD4 PE
CD62L APC-Cy7
CD44 PerCP-Cy5.5
CD45.1 BV605
OVA pentamer APC
Counts
CellTrace Violet
b
LNs
OVA alone
OVA + CD8α ALN-1
CD8 APC
CD45.1 BV605
x 2.5
OT-I in LNs
OVA alone
OVA + CD8α ALN-1
ns
p = 0.0556
CD45.1+ cells (% of all CD8+ T cells)
CD45.1+ cells (counts in CD8+ T cells)
Relative
Absolute
c
Spleen
OVA alone
OVA + CD8α ALN-1
CD8 APC
CD45.1 BV605
x 3.6
OT-I in spleen
OVA alone
OVA + CD8α ALN-1
*
*
Relative
Absolute
d
Lung parenchyma
OVA alone
OVA + CD8α ALN-1
CD8 APC
CD45.1 BV605
x 5.2
CD45.1 BV605
OT-I in lung
OVA alone
OVA + CD8α ALN-1
*
p = 0.0635
Relative
Absolute
e
Liver
OVA alone
OVA + CD8α ALN-1
CD8 APC
CD45.1 BV605
x 12.9
OT-I in liver
OVA alone
OVA + CD8α ALN-1
p = 0.0952
*
Relative
Absolute
f
Spleen
PBS
OVA alone
OVA + LPS
OVA + WT IL-1β
OVA + CD8α ALN-1
OVA + Bcl10 ALN-1
CD62L APC-Cy7
CD44 PerCP-Cy5.5

Supplementary Figure 4. a. Gating strategy for the detection of CTL activation and OT-I proliferation after transfer in C57BL/6 (IL-1R1$^{-/-}$) recipient mice. Staining of splenocytes with CD19, CD3, CD4, CD62L, CD44 and CD45.1 antibodies and OVA-pentamer. **b – e.** Immunization with OVA and CD8α ALN-1 promotes OT-I proliferation and increases the amount of OT-I cells in lymphoid and peripheral tissues compared with OVA treatment alone. Experiment performed according to Fig. 3a with two treatment groups: OVA (100 μg i.p.) alone or combined with CD8α ALN-1 (10 μg i.p. daily for three consecutive days). Staining with LIVE/DEAD dye and CD19, CD3, CD4, CD8 APC (250x dilution) (clone 53-6.7, 553035, BioLegend) and CD45.1 antibodies. OT-I cells were detected in the single cell population (based on FSC/SSC) as LIVE/DEAD^{-}CD19^{-}CD3^{+}CD4^{-}CD8^{+}CD45.1^{+} cells. Displayed are the relative (% in the CD8^{+} T cell population) and absolute (counts in the CD8^{+} T cell population) amounts of OT-I cells in LNs (**b**), spleen (**c**), lungs (**d**) and liver (**e**). Bars represent the mean ± s.e.m. of one experiment with $n = 5$ mice/group. *, $p < 0.05$; ns, $p \geq 0.05$ by unpaired Mann-Whitney U test (two-tailed). **f.** Representative dot plots illustrating simultaneous upregulation of CD44 and downregulation of CD62L on CTLs upon treatment with OVA and CD8α ALN-1. Controls include treatment without antigen (PBS) and with OVA alone or combined with either LPS, WT IL-1β or BcII10 ALN-1.

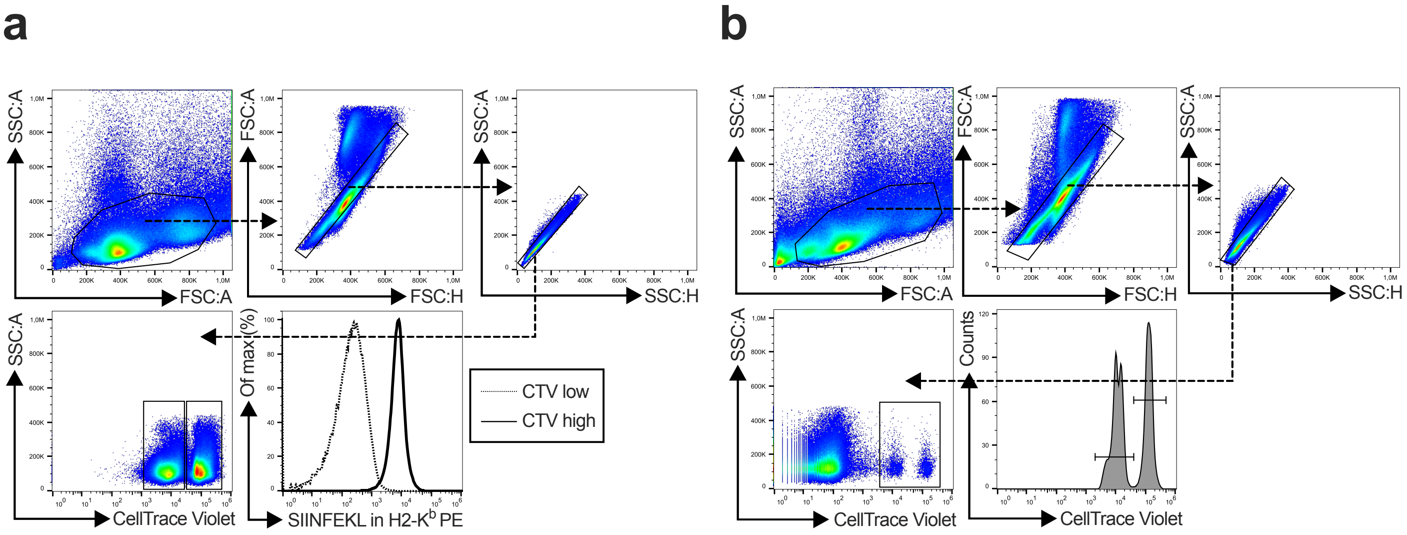

a
b
SSC:A
FSC:A
FSC:H
SSC:H
CellTrace Violet
Of max (%)
SIINFEKL in H2-K^b PE
CTV low
CTV high
Counts

Supplementary Figure 5. a. Splenocyte labeling for the *in vivo* killing assay experiment. Cells were labeled with a high or low intensity of CTV and loaded with SIINFEKL (CTV^{high}) or left unloaded (CTV^{low}). Peptide presentation is detected with a SIINFEKL in H2-K^b antibody. **b.** Gating strategy for the detection of adoptively transferred target cells in the spleens of acceptor mice.

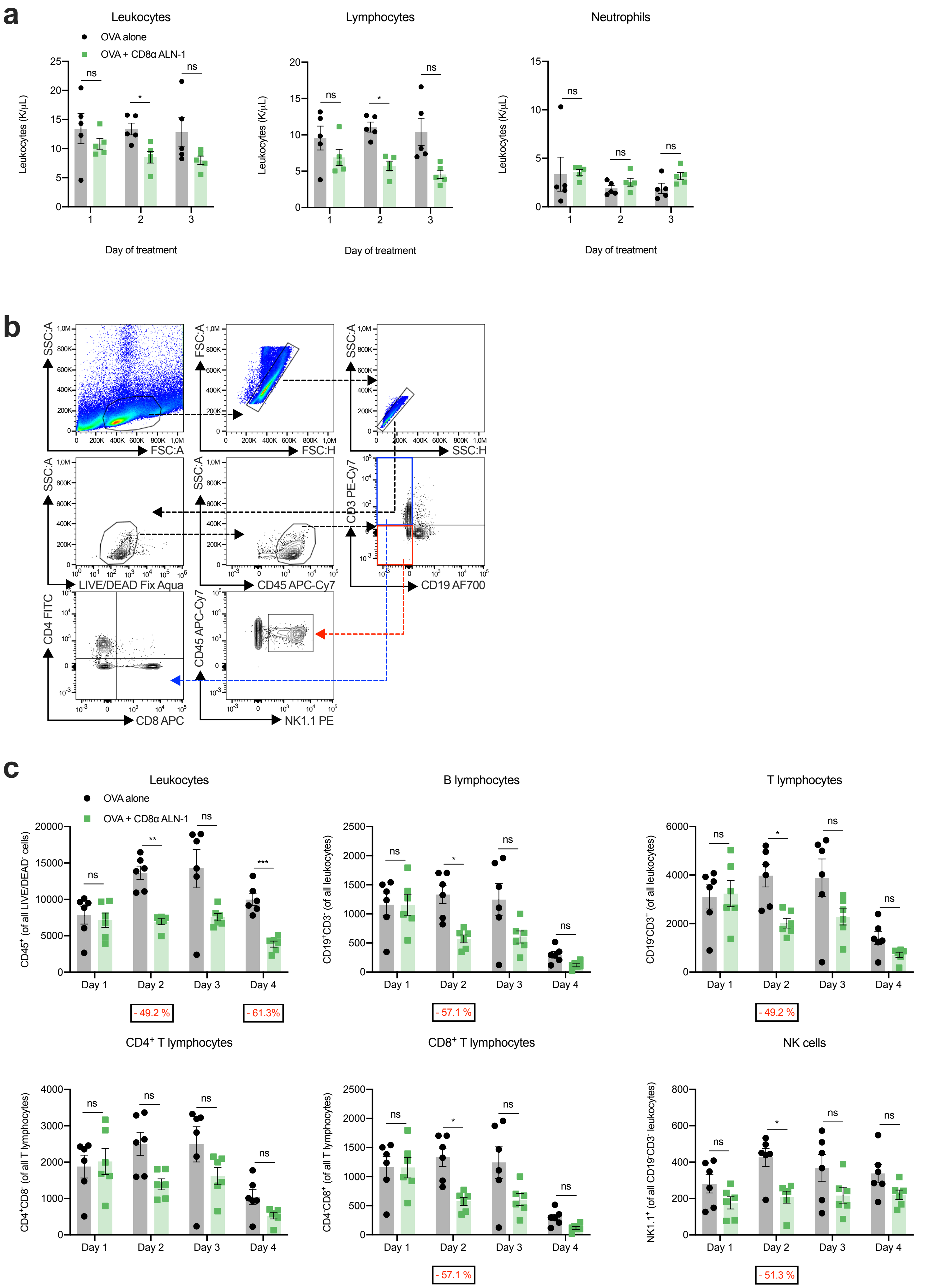
a
Leukocytes
OVA alone
OVA + CD8α ALN-1
Leukocytes (K/μL)
Day of treatment
Lymphocytes
Leukocytes (K/μL)
Day of treatment
Neutrophils
Leukocytes (K/μL)
Day of treatment
b
SSC:A
FSC:A
FSC:A
FSC:H
SSC:A
SSC:H
SSC:A
LIVE/DEAD Fix Aqua
SSC:A
CD45 APC-Cy7
CD3 PE-Cy7
CD19 AF700
CD4 FITC
CD8 APC
CD45 APC-Cy7
NK1.1 PE
c
Leukocytes
OVA alone
OVA + CD8α ALN-1
CD45+ (of all LIVE/DEAD- cells)
- 49.2 %
- 61.3%
B lymphocytes
CD19+CD3- (of all leukocytes)
- 57.1 %
T lymphocytes
CD19-CD3+ (of all leukocytes)
- 49.2 %
CD4+ T lymphocytes
CD4+CD8- (of all T lymphocytes)
CD8+ T lymphocytes
CD4-CD8+ (of all T lymphocytes)
- 57.1 %
NK cells
NK1.1+ (of all CD19-CD3- leukocytes)
- 51.3 %
Day 1
Day 2
Day 3
Day 4

Supplementary Figure 6. a. C57BL/6 mice were treated i.p. with OVA (100 μg), either alone or combined with CD8α ALN-1 (daily i.p. treatment with 10 μg for three consecutive days). Tail vein blood was sampled 6h after every administration and analyzed on the Hemavet 950FS system. Bars represent the mean ± s.e.m. of one experiment with $n = 5$ mice/group. **b.** Gating strategy for the detection of different lymphocyte populations in mouse peripheral blood. Staining with LIVE/DEAD dye and CD45, CD19, CD3, CD4, CD8 and NK1.1 antibodies. **c.** Flow cytometry analysis for measurement of absolute cells counts in mouse peripheral blood, sampled at the indicated timepoints as described in (**a**). Day 4 means sampling 24h after the last treatment. In the single (based on FSC/SSC) living (LIVE/DEAD^{-}) cell population, we identified leukocytes (CD45^{+}), B cells (CD45^{+}CD19^{+}CD3^{-}), T cells (CD45^{+}CD19^{-}CD3^{+}), CD4^{+} T cells (CD45^{+}CD19^{-}CD3^{+}CD4^{+}CD8^{-}), CD8^{+} T cells (CD45^{+}CD19^{-}CD3^{+}CD4^{-}CD8^{+}) and NK cells (CD45^{+}CD19^{-}CD3^{-}NK1.1^{+}). Bars represent the mean ± s.e.m. of one experiment with $n = 6$ mice/group. ***, $p < 0.001$; **, $p < 0.01$; *, $p < 0.05$; ns, $p \geq 0.05$ by two-way repeated measures ANOVA with Sidak's multiple comparisons test in (**a**) and (**c**).

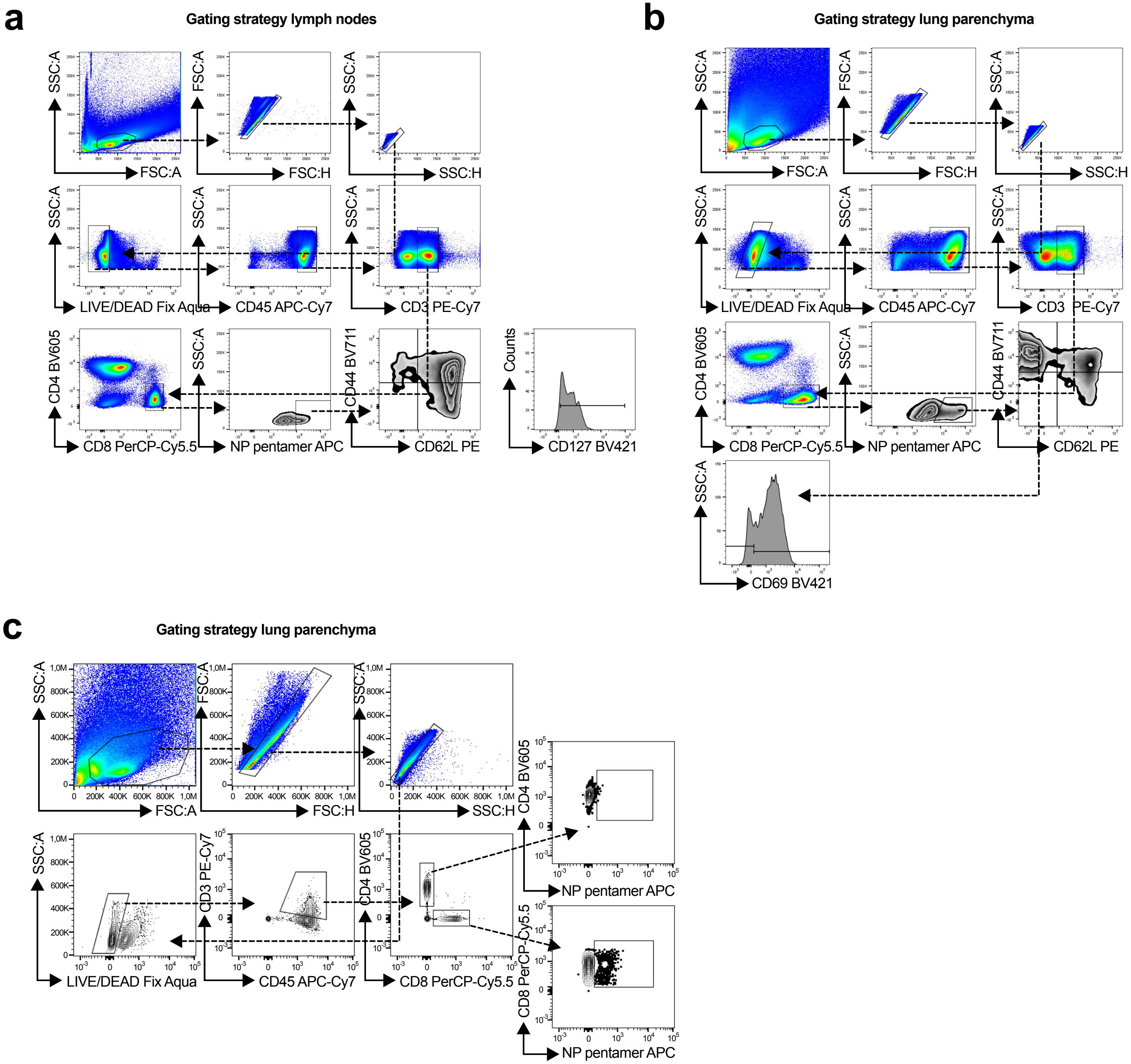
a
Gating strategy lymph nodes
SSC:A
FSC:A
FSC:H
SSC:H
LIVE/DEAD Fix Aqua
CD45 APC-Cy7
CD3 PE-Cy7
CD4 BV605
CD8 PerCP-Cy5.5
NP pentamer APC
CD44 BV711
CD62L PE
Counts
CD127 BV421
b
Gating strategy lung parenchyma
SSC:A
FSC:A
FSC:H
SSC:H
LIVE/DEAD Fix Aqua
CD45 APC-Cy7
CD3 PE-Cy7
CD4 BV605
CD8 PerCP-Cy5.5
NP pentamer APC
CD44 BV711
CD62L PE
CD69 BV421
c
Gating strategy lung parenchyma
SSC:A
FSC:A
FSC:H
SSC:H
LIVE/DEAD Fix Aqua
CD3 PE-Cy7
CD45 APC-Cy7
CD4 BV605
CD8 PerCP-Cy5.5
NP pentamer APC

Supplementary Figure 7. a and b. Gating strategy for the detection of NP-specific CTLs in lung-draining mediastinal LNs (**a**) or lung parenchyma (**b**) one week *post*-influenza infection. Staining with LIVE/DEAD dye, CD45, CD3, CD4, CD8, CD44, CD62L, CD127 and CD69 antibodies and NP-pentamer. **c.** Gating strategy for the detection of long-lasting NP-specific CTLs in lung parenchyma 50 days *post*-influenza infection. Staining with LIVE/DEAD dye, CD45, CD3, CD4 and CD8 antibodies and NP-pentamer. Panels on the right indicate the cleanliness of the NP pentamer staining, which is shown to be selective for $CD8^+$ T cells.

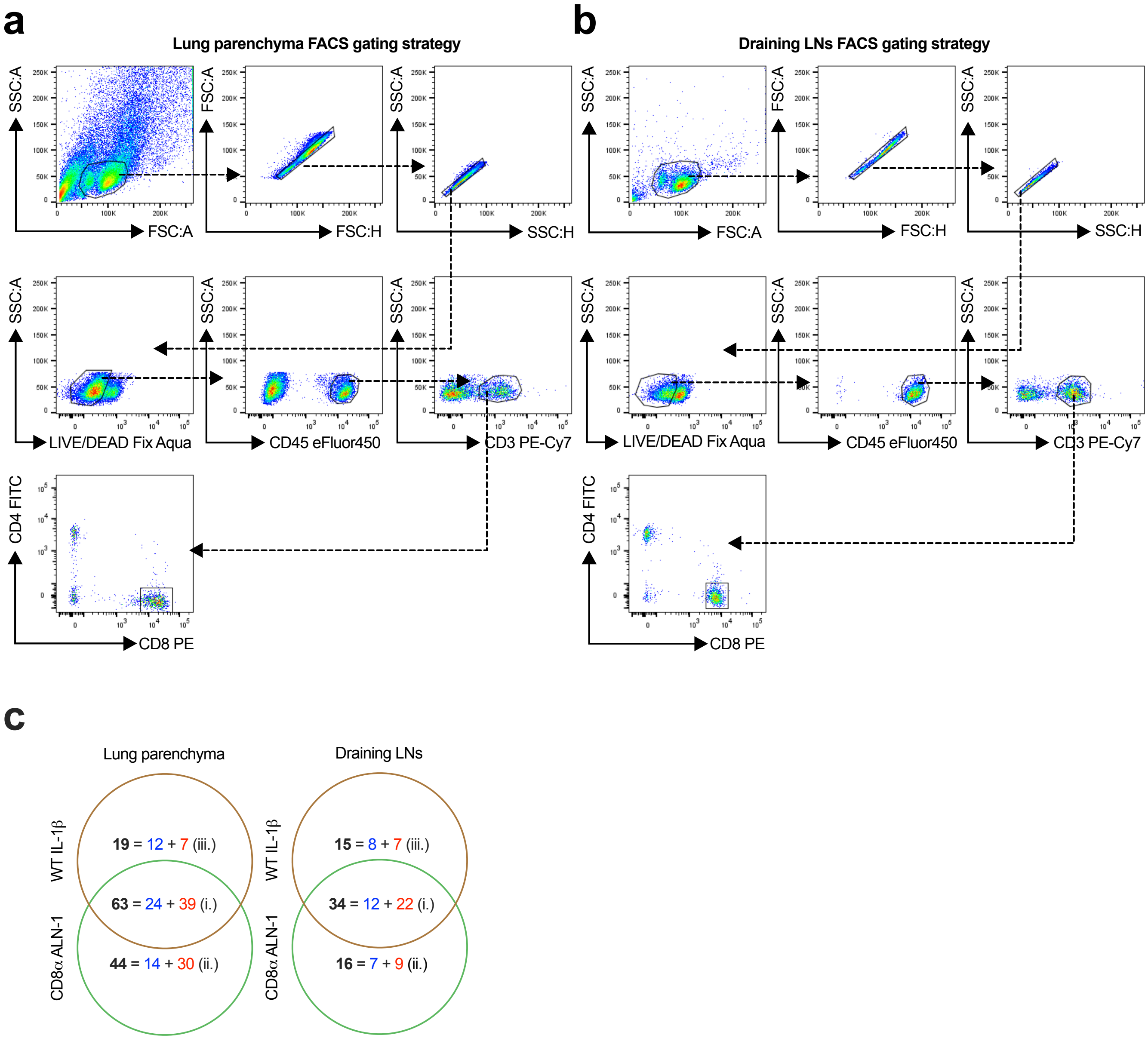
a
Lung parenchyma FACS gating strategy
b
Draining LNs FACS gating strategy
SSC:A
FSC:A
FSC:H
SSC:H
LIVE/DEAD Fix Aqua
CD45 eFluor450
CD3 PE-Cy7
CD4 FITC
CD8 PE
c
Lung parenchyma
Draining LNs
WT IL-1β
CD8α ALN-1
19 = 12 + 7 (iii.)
63 = 24 + 39 (i.)
44 = 14 + 30 (ii.)
15 = 8 + 7 (iii.)
34 = 12 + 22 (i.)
16 = 7 + 9 (ii.)

Supplementary Figure 8. Gating strategy for sorting $CD8^+$ T lymphocytes from the lung parenchyma (**a**) and lung-draining LNs (**b**) of influenza-infected mice one week *post*-influenza A virus challenge. Staining with LIVE/DEAD dye and CD45, CD3, CD4 and CD8 antibodies. **c.** Venn diagrams representing the numbers of DEGs shared between (i.) or unique for treatment with WT IL-1β (iii.) and/or CD8α ALN-1 (ii.). Upregulated genes are printed in blue, whereas downregulated genes are printed in red. In $CD8^+$ T cells isolated from lung, 63 differentially expressed genes (DEGs) (24 up- and 39 downregulated) were shared between WT IL-1β and CD8α ALN-1-vaccinated mice. Both WT IL-1β and CD8α ALN-1 had non-redundant effects on gene modulation in lung, as evidenced by 19 DEGs (12 up- and 7 downregulated) unique to WT IL-1β and 44 DEGs (14 up- and 30 downregulated) unique to CD8α ALN-1 treatment. In $CD8^+$ T cells sorted from draining LNs, 34 DEGs (12 up- and 22 downregulated) were shared between mice that received WT IL-1β or CD8α ALN-1 as adjuvant. Here, we identified 15 DEGs (8 up- and 7 downregulated) modulated by WT IL-1β only and 16 DEGs (7 up- and 9 downregulated) uniquely regulated upon CD8α ALN-1-treatment.

Supplementary Table 1. Overview of the used primers for RT-qPCR analysis.

Gene	Forward primer (5'-3')	Reverse primer (5'-3')
IL8	CTCTCTTGGCAGCCTTCCTGA	CAATAATTTCTGTGTTGGCGC
NFKBIA	CTCCGAGACTTTCGAGGAAATAC	GCCATTGTAGTTGGTAGCCTTCA
A20	CCTTGCTTTGAGTCAGGCTGT	AAGGAGAAGCACGAAACATC
JUN	CAGGTGGCACAGCTTAAACA	GTTTGCAACTGCTGCGTTAG
DUSP	CTGCCTTGATCAACGTCTCA	CTGTGCCTTGTGGTTGTCCT
ICAM1	GCAGACAGTGACCATCTACAGCTT	CTTCTGAGACCTCTGGCTTCGT
HPRT	TGACACTGGCAAAACAATGCA	GGTCCTTTTCACCAGCAAGCT

Supplementary Table 2. Overview of all DEGs identified in lung and lung-draining LNs upon vaccination with WIV and WT IL-1β or CD8α ALN-1, compared with mice vaccinated with WIV alone.

ENSEMBL Gene_ID	Gene name	WIV + WT IL-1β			WT + CD8α ALN-1		
		log2FC	*p*-value	DE	log2FC	*p*-value	DE
**Lung parenchyma**							
ENSMUSG00000019987	Arg1	4,05711509	1,98608E-17	1	4,29590228	2,93394E-19	1
ENSMUSG00000022912	Pros1	2,5996262	8,11367E-18	1	2,39043136	1,93275E-15	1
ENSMUSG00000009185	Ccl8	2,25720213	3,77028E-11	1	2,63395779	1,51232E-14	1
ENSMUSG00000033965	Slc16a2	3,29686108	1,51687E-08	1	3,36672083	7,94177E-09	1
ENSMUSG00000052336	Cx3cr1	0,86365501	0,005162968	0	1,76422628	1,05687E-08	1
ENSMUSG00000014599	Csf1	2,2351707	3,47146E-06	1	2,75627883	1,77563E-08	1
ENSMUSG00000055865	Fam19a3	2,75657559	5,69926E-11	1	2,25778529	4,88286E-08	1
ENSMUSG00000029359	Tesc	0,96307008	8,53673E-06	0	1,12498886	1,66132E-07	1
ENSMUSG00000079037	Prnp	1,7398404	3,04475E-11	1	1,36337326	1,79126E-07	1
ENSMUSG00000030352	Tspan9	2,16707305	5,73319E-08	1	2,05959273	2,26297E-07	1
ENSMUSG00000029304	Spp1	3,02792241	6,98512E-05	1	4,02811783	4,23329E-07	1
ENSMUSG00000040809	Chil3	3,77836836	2,84377E-05	1	4,76388476	4,37864E-07	1
ENSMUSG00000039911	Spsb1	1,13324042	1,00843E-07	1	1,03588277	1,07963E-06	1
ENSMUSG00000042262	Ccr8	3,4415482	5,39781E-05	1	4,23286469	1,45142E-06	1
ENSMUSG00000032204	Aqp9	1,6685762	2,49278E-07	1	1,54636116	1,65235E-06	1
ENSMUSG00000030669	Calca	1,13512733	0,000910187	1	1,59391536	2,02368E-06	1
ENSMUSG00000042359	Osbpl6	1,31840417	2,27917E-09	1	1,02457807	3,23103E-06	1
ENSMUSG00000061100	Retnla	3,16166848	1,10924E-08	1	2,54793697	3,42425E-06	1
ENSMUSG00000045573	Penk	2,2632106	0,002486791	0	3,45687142	6,81887E-06	1
ENSMUSG00000029075	Tnfrsf4	1,42798331	0,000198875	1	1,72891258	7,48657E-06	1
ENSMUSG00000099413	Gm17767	1,60137183	7,9107E-08	1	1,23661683	3,22804E-05	1
ENSMUSG00000052117	D630039A03Rik	2,21225138	3,66814E-06	1	1,95147548	4,03817E-05	1
ENSMUSG0000029120	Ppp2r2c	1,71771823	0,008234809	0	2,72549744	4,38767E-05	1
ENSMUSG00000019122	Ccl9	1,66200345	0,001373433	0	2,12851347	5,00545E-05	1
ENSMUSG00000055435	Maf	0,61138363	0,043916574	0	1,22278161	5,39262E-05	1
ENSMUSG00000029153	Ociad2	1,92444424	2,35004E-05	1	1,77497234	9,16908E-05	1
ENSMUSG00000049410	Zfp683	1,7741495	0,002701268	0	2,3042335	0,000103513	1
ENSMUSG00000020702	Ccl1	2,88039173	0,000290566	1	3,09514944	0,00011028	1
ENSMUSG00000028159	Dapp1	1,2210735	1,36519E-12	1	0,86660649	4,25477E-07	0
ENSMUSG00000024873	Cnih2	0,79937102	0,014914871	0	1,24340553	0,000139325	1
ENSMUSG00000036067	Slc2a6	2,03424683	0,00077107	1	2,25615002	0,000198519	1
ENSMUSG00000095105	Edaradd	0,58204527	0,106138157	0	1,30111476	0,000230131	1

ENSMUSG00000024891	Slc29a2	1,08639527	0,000457067	1	1,08159868	0,000425383	1
ENSMUSG00000032122	Slc37a2	0,70978204	0,015427195	0	1,02315636	0,000476749	1
ENSMUSG00000030257	Srgap3	1,56216686	0,001013261	1	1,59815768	0,000754643	1
ENSMUSG00000047751	Utf1	0,87119172	0,056711602	0	1,52058241	0,000833388	1
ENSMUSG00000026841	Fibcd1	0,81427195	0,074615887	0	1,50845181	0,000850104	1
ENSMUSG00000053310	Nrgn	1,40054857	0,038368344	0	2,26662369	0,000948457	1
ENSMUSG00000076474	Trbv17	1,35625214	0,004418586	0	1,5682474	0,001055005	1
ENSMUSG00000037820	Tgm2	1,45826264	1,44706E-09	1	0,90985835	0,000144036	0
ENSMUSG00000040848	Sft2d2	1,03637637	2,19136E-09	1	0,76229809	9,97189E-06	0
ENSMUSG00000041762	Gpr155	1,14979823	8,7733E-09	1	0,93935052	2,48216E-06	0
ENSMUSG00000076757	Tcrg-C4	1,58381663	1,53385E-07	1	0,73692229	0,017634392	0
ENSMUSG00000030748	Il4ra	1,10362954	4,56966E-07	1	0,62094863	0,004229953	0
ENSMUSG00000049985	Ankrd55	1,29894741	1,98788E-05	1	0,90390611	0,003133471	0
ENSMUSG00000049555	Tmie	1,01932981	0,000104474	1	0,5844977	0,02576124	0
ENSMUSG00000090958	Lrrc32	1,76900216	0,00019792	1	1,39752827	0,003388024	0
ENSMUSG00000103847	Gm20056	1,07489849	0,000239112	1	0,48936367	0,107354048	0
ENSMUSG00000024653	Scgb1a1	1,27599674	0,000430123	1	0,66349799	0,069011614	0
ENSMUSG00000044068	Zrsr1	1,07554764	0,000462105	1	0,93732562	0,002282321	0
ENSMUSG00000026003	Acadl	-1,13342	2,56996E-09	-1	-0,9413783	6,67391E-07	0
ENSMUSG00000037731	Themis2	-1,06868	1,47542E-05	-1	-0,8920107	0,000262705	0
ENSMUSG00000036882	Arhgap33	-1,0462149	4,61725E-05	-1	-0,355519	0,14784419	0
ENSMUSG00000034947	Tmem106a	-1,0159908	4,94035E-05	-1	-0,7532037	0,002311443	0
ENSMUSG00000030786	Itgam	-1,4346074	8,40408E-05	-1	-1,0021713	0,00546377	0
ENSMUSG00000020389	Cdkl3	-1,121136	0,00013493	-1	-0,9181567	0,001520632	0
ENSMUSG00000069662	Marcks	-1,0794951	0,000643674	-1	-0,9001995	0,003977419	0
ENSMUSG00000002307	Daxx	-0,9641876	1,25396E-10	0	-1,3716882	1,2665E-19	-1
ENSMUSG00000025498	Irf7	-0,8113792	2,58602E-10	0	-1,0400238	7,28404E-16	-1
ENSMUSG00000030966	Trim21	-0,7979276	1,85679E-08	0	-1,1075851	8,99017E-15	-1
ENSMUSG00000027514	Zbp1	-0,9168034	5,76558E-09	0	-1,1748818	1,14271E-13	-1
ENSMUSG00000060519	Tor3a	-0,9737058	4,49754E-09	0	-1,2113253	4,16894E-13	-1
ENSMUSG00000035208	Slfn8	-0,9413618	4,70562E-07	0	-1,3576018	6,20358E-13	-1
ENSMUSG00000069874	Irgm2	-0,693458	1,42838E-06	0	-1,0256472	1,37708E-12	-1
ENSMUSG00000027078	Ube2l6	-0,7735168	4,69082E-05	0	-1,3416154	4,8373E-12	-1
ENSMUSG00000046879	Irgm1	-0,7415023	4,33445E-06	0	-1,0999635	1,28939E-11	-1
ENSMUSG00000043263	Pyhin1	-0,9393559	8,8686E-09	0	-1,1038808	1,67454E-11	-1
ENSMUSG00000028037	Ifi44	-2,4198171	1,45743E-16	-1	-2,8148523	4,27672E-21	-1
ENSMUSG00000032661	Oas3	-0,9133908	3,63876E-08	0	-1,0574046	2,04254E-10	-1
ENSMUSG00000078763	Slfn1	-0,8274738	2,51882E-07	0	-1,0108995	3,4441E-10	-1
ENSMUSG00000043953	Ccrl2	-0,9895467	7,41914E-07	0	-1,2243972	1,13841E-09	-1
ENSMUSG00000021356	Irf4	-0,7174509	0,001107453	0	-1,3176557	3,30337E-09	-1
ENSMUSG00000069892	9930111J21Rik2	-0,9531864	1,38148E-07	0	-1,0360355	1,07752E-08	-1
ENSMUSG00000066677	Pydc3	-0,7603448	1,78457E-05	0	-1,0181661	1,08409E-08	-1

ENSMUSG00000033355	Rtp4	-1,0899875	3,92515E-12	-1	-1,4672138	2,23446E-20	-1
ENSMUSG00000039697	Ncoa7	-0,8091121	0,000593776	0	-1,3547897	1,37087E-08	-1
ENSMUSG00000006345	Ggt1	-0,8197185	1,92304E-05	0	-1,0872046	1,72151E-08	-1
ENSMUSG00000017830	Dhx58	-0,942323	1,60569E-05	0	-1,217792	3,05952E-08	-1
ENSMUSG00000078349	AW011738	-0,8776424	2,41219E-05	0	-1,1539659	3,36404E-08	-1
ENSMUSG00000045932	Ifit2	-1,2833196	4,38028E-08	-1	-1,9552511	3,08185E-16	-1
ENSMUSG00000091144	Phf11c	-1,3419377	5,48995E-13	-1	-1,4994502	1,02011E-15	-1
ENSMUSG00000036381	P2ry14	-0,7455419	0,001679001	0	-1,3158071	5,02145E-08	-1
ENSMUSG00000070501	BC094916	-0,8263604	1,57952E-05	0	-1,037525	6,83083E-08	-1
ENSMUSG00000094796	BC147527	-0,912573	5,90552E-05	0	-1,2014109	1,62597E-07	-1
ENSMUSG00000082292	Gm12250	-0,4509113	0,019872406	0	-1,005211	2,6695E-07	-1
ENSMUSG00000069893	9930111J21Rik1	-0,8661951	7,00369E-05	0	-1,0703578	9,92518E-07	-1
ENSMUSG00000029605	Oas1b	-0,6308381	0,00775449	0	-1,0552358	1,05748E-05	-1
ENSMUSG00000073491	Pydc4	-0,9410605	8,37799E-05	0	-1,0288438	1,79793E-05	-1
ENSMUSG00000039236	Isg20	-1,3483339	8,18696E-12	-1	-1,5425013	7,16068E-15	-1
ENSMUSG00000054404	Slfn5	-1,4905379	2,79577E-07	-1	-2,3153528	8,72872E-15	-1
ENSMUSG00000044734	Serpinb1a	-0,7717839	0,001697613	0	-1,0180339	3,97168E-05	-1
ENSMUSG00000035517	Tdrd7	-0,6408535	0,019132996	0	-1,0513343	0,000151456	-1
ENSMUSG00000026880	Stom	-0,8847861	0,002124242	0	-1,0570212	0,000256837	-1
ENSMUSG00000023341	Mx2	-1,651011	1,32111E-06	-1	-2,6776885	4,42714E-14	-1
ENSMUSG00000020638	Cmpk2	-1,7704884	3,03101E-11	-1	-2,0251802	4,8981E-14	-1
ENSMUSG00000079017	Ifi27l2a	-1,5155727	4,15276E-13	-1	-1,577349	4,9091E-14	-1
ENSMUSG00000030107	Usp18	-1,0034437	3,43408E-07	-1	-1,4713893	1,4993E-13	-1
ENSMUSG00000034459	Ifit1	-1,7311148	9,52699E-08	-1	-2,453831	1,68862E-13	-1
ENSMUSG00000034855	Cxcl10	-1,4544659	4,03181E-09	-1	-1,7707311	1,55693E-12	-1
ENSMUSG00000035692	Isg15	-1,2879742	2,13371E-07	-1	-1,7602272	2,75438E-12	-1
ENSMUSG00000026896	Ifih1	-1,1279316	8,72075E-08	-1	-1,4874444	2,80709E-12	-1
ENSMUSG00000040483	Xaf1	-1,0156472	3,33222E-09	-1	-1,1906955	4,97549E-12	-1
ENSMUSG00000018986	Slfn3	-1,2641461	3,6997E-08	-1	-1,5666263	1,56405E-11	-1
ENSMUSG00000074896	Ifit3	-1,4938574	3,51448E-06	-1	-2,2207335	1,71259E-11	-1
ENSMUSG00000091649	Phf11b	-1,1186913	1,94135E-12	-1	-1,0513731	3,268E-11	-1
ENSMUSG00000025743	Sdc3	-1,1899111	8,39715E-09	-1	-1,3523276	6,94628E-11	-1
ENSMUSG00000041827	Oasl1	-1,7489338	2,54327E-06	-1	-2,4644078	1,10655E-10	-1
ENSMUSG00000000386	Mx1	-1,647277	7,28894E-08	-1	-1,9922848	1,22035E-10	-1
ENSMUSG00000062488	Ifit3b	-1,9742997	1,16064E-06	-1	-2,5263631	1,1823E-09	-1
ENSMUSG00000067297	Ifit1bl2	-1,4000766	7,17677E-05	-1	-2,2155706	1,43437E-09	-1
ENSMUSG00000037849	Gm4955	-1,2209231	1,23405E-06	-1	-1,5132802	2,4825E-09	-1
ENSMUSG00000020641	Rsad2	-1,6814335	2,03892E-05	-1	-2,3977183	3,2054E-09	-1
ENSMUSG00000052776	Oas1a	-1,1907072	1,91488E-06	-1	-1,4233934	1,51428E-08	-1
ENSMUSG00000032690	Oas2	-1,2338848	0,000238711	-1	-1,803155	1,29475E-07	-1
ENSMUSG00000021190	Lgmn	-1,5710594	1,24926E-08	-1	-1,4417479	1,3868E-07	-1
ENSMUSG00000035493	Tgfbi	-2,0602198	6,7455E-07	-1	-2,1202142	3,27653E-07	-1

ENSMUSG00000079339	Ifit1bl1	-1,5850687	0,00019635	-1	-2,1493129	7,22534E-07	-1
ENSMUSG00000078616	Trim30c	-1,3606698	4,45107E-06	-1	-1,4611364	8,75847E-07	-1
ENSMUSG00000044703	Phf11a	-1,7046824	1,36514E-09	-1	-1,345235	1,2163E-06	-1
ENSMUSG00000024079	Eif2ak2	-1,1697848	6,01846E-05	-1	-1,4191296	1,30396E-06	-1
ENSMUSG00000049608	Gpr55	-1,0942929	0,000401493	-1	-1,4379794	4,17759E-06	-1
ENSMUSG00000032359	Ctsh	-1,095481	3,06596E-05	-1	-1,1013949	2,61715E-05	-1
ENSMUSG00000026535	Ifi202b	-1,1975888	0,00098766	-1	-1,5168638	3,72111E-05	-1
ENSMUSG00000075602	Ly6a	-1,1800157	8,53629E-05	-1	-1,197081	6,76324E-05	-1
ENSMUSG00000025492	Ifitm3	-1,4283248	0,000581796	-1	-1,5599241	0,000181309	-1
ENSMUSG00000003545	Fosb	-1,7769018	4,37802E-06	-1	-1,4046676	0,000235493	-1
**Lung-draining mediastinal lymph nodes**							
ENSMUSG00000003545	Fosb	-3,0817279	2,91242E-13	-1	-3,3189305	6,13461E-15	-1
ENSMUSG00000076614	Ighg1	4,17482229	5,21065E-10	1	5,34392845	4,90133E-14	1
ENSMUSG00000003032	Klf4	-1,8340445	2,41405E-09	-1	-1,9679008	1,67025E-10	-1
ENSMUSG00000021250	Fos	-1,1416387	1,26382E-06	-1	-1,4453601	8,84448E-10	-1
ENSMUSG00000054364	Rhob	-0,8091276	2,09877E-06	0	-1,02952	1,64571E-09	-1
ENSMUSG00000033355	Rtp4	-1,0302063	2,39478E-09	-1	-1,0172309	3,766E-09	-1
ENSMUSG00000028037	Ifi44	-1,8596369	6,2858E-09	-1	-1,800561	1,65708E-08	-1
ENSMUSG00000027078	Ube2l6	-0,8548343	7,3861E-05	0	-1,1981519	3,53221E-08	-1
ENSMUSG00000026628	Atf3	-0,8026744	0,00234032	0	-1,471968	4,80296E-08	-1
ENSMUSG00000065922	n-R5-8s1	-1,6187259	7,05121E-06	-1	-1,9706953	5,6767E-08	-1
ENSMUSG00000039236	Isg20	-1,1998699	8,56547E-08	-1	-1,1947154	9,16149E-08	-1
ENSMUSG00000042622	Maff	-0,9266496	8,97217E-06	0	-1,1095909	1,07989E-07	-1
ENSMUSG00000062593	Lilrb4a	1,63827098	2,13744E-06	1	1,5781239	4,94384E-06	1
ENSMUSG00000037849	Gm4955	-1,1089818	6,10502E-05	-1	-1,2633452	4,99905E-06	-1
ENSMUSG00000034459	Ifit1	-1,3323291	0,000145901	-1	-1,5689792	8,0168E-06	-1
ENSMUSG00000017830	Dhx58	-0,9048185	0,000182024	0	-1,032352	1,96258E-05	-1
ENSMUSG00000076613	Ighg2b	0,96920787	0,084325298	0	2,49722694	2,66664E-05	1
ENSMUSG00000075602	Ly6a	-1,52039	3,43703E-06	-1	-1,3512502	3,6224E-05	-1
ENSMUSG00000034855	Cxcl10	-1,1786243	9,42142E-06	-1	-1,0725535	5,36259E-05	-1
ENSMUSG00000022912	Pros1	1,80960591	8,4399E-07	1	1,44135199	8,28765E-05	1
ENSMUSG00000057329	Bcl2	1,38018861	6,10313E-07	1	1,07130406	8,82366E-05	1
ENSMUSG00000074896	Ifit3	-1,2649294	0,000336824	-1	-1,3754694	9,81573E-05	-1
ENSMUSG00000076522	Igkv16-104	0,78020131	0,448734986	0	3,86908725	0,000122915	1
ENSMUSG00000033965	Slc16a2	2,53569445	0,000101252	1	2,43925688	0,000173491	1
ENSMUSG00000097971	Gm26917	-1,3212673	0,00061325	-1	-1,4422058	0,000185952	-1
ENSMUSG00000020882	Cacnb1	1,10538898	4,36299E-05	1	1,00889465	0,000186842	1
ENSMUSG00000085028	Slc2a4rg-ps	1,0986135	6,86719E-05	1	1,02731196	0,000190872	1
ENSMUSG00000097061	9330151L19Rik	1,17699018	5,87438E-05	1	1,0863881	0,000202039	1
ENSMUSG00000103593	Gm37352	-0,8906948	0,004289023	0	-1,1353236	0,000281685	-1
ENSMUSG00000089672	Lilr4b	1,8496286	1,47315E-06	1	1,37654361	0,000303076	1
ENSMUSG00000020638	Cmpk2	-1,1484957	7,52954E-05	-1	-1,0412255	0,000324891	-1

ENSMUSG00000037465	Klf10	-1,1079968	6,52887E-06	-1	-0,8820539	0,000330695	0
ENSMUSG00000085396	Firre	1,54235327	8,31202E-05	1	1,40252168	0,000362088	1
ENSMUSG00000067149	Jchain	1,645183	0,011916432	0	2,38764376	0,000418234	1
ENSMUSG00000076934	Iglv1	1,08163975	0,106720361	0	2,3987837	0,000448506	1
ENSMUSG00000076612	Ighg2c	2,13683457	0,00957009	0	2,99560755	0,000506888	1
ENSMUSG00000044583	Tlr7	-1,0373085	0,001392533	0	-1,1147491	0,000610562	-1
ENSMUSG00000092274	Neat1	1,31316326	0,001174731	0	1,38087598	0,000668034	1
ENSMUSG00000032204	Aqp9	1,27676229	0,00060216	1	1,19612354	0,001286767	0
ENSMUSG00000014932	Yes1	1,13397374	2,70791E-05	1	0,85677188	0,001593291	0
ENSMUSG00000023348	Trip6	1,65309047	0,000225556	1	1,40282877	0,001669877	0
ENSMUSG00000030651	Art2b	1,03880844	0,000341106	1	0,88424178	0,002197629	0
ENSMUSG00000058773	Hist1h1b	-1,6806687	0,000359909	-1	-1,3747913	0,003298826	0
ENSMUSG00000026285	Pdcd1	-1,2247041	0,000283558	-1	-0,9574842	0,004450772	0
ENSMUSG00000044068	Zrsr1	1,48617691	3,89184E-05	1	0,9881265	0,006178052	0
ENSMUSG00000028341	Nr4a3	-1,0015257	5,87175E-05	-1	-0,6580178	0,008364033	0
ENSMUSG00000015437	Gzmb	-1,5988982	0,0006972	-1	-1,1899122	0,011468658	0
ENSMUSG00000025350	Rdh5	1,5759155	0,000127307	1	0,91061354	0,028734819	0
ENSMUSG00000052736	Klrc2	-1,231146	0,000792997	-1	-0,7669356	0,035268974	0
ENSMUSG00000108132	RP23-322E20.8	1,04689159	0,000436622	1	0,57982271	0,050192788	0
ENSMUSG00000020841	Cpd	-1,4410335	0,000702326	-1	-0,6507141	0,121780333	0
